# Uncertainty about the risks associated with microplastics among lay and topic-experienced respondents

**DOI:** 10.1038/s41598-021-86569-5

**Published:** 2021-03-30

**Authors:** Christina J. Thiele, Malcolm D. Hudson

**Affiliations:** Centre for Environmental Science, Faculty of Environment and Life Sciences, University of Southampton, University Road, Southampton, SO17 1BJ UK

**Keywords:** Environmental sciences, Environmental social sciences

## Abstract

Microplastics are contaminants of emerging concern but there is currently a lack of evidence on actual risks relating to them, despite claims in media and scientific articles. Research on people’s perceptions on microplastics is in its infancy. Here we present part of a larger survey about people’s perceptions of issues with microplastics. Our analysis of 1681 responses across the globe to an online questionnaire demonstrates a certain level of uncertainty, not only in lay people but also respondents who study/work on the topic of plastics and microplastics as a pollutant. This uncertainty ranges from level of concern about microplastics as an environmental issue to existing evidence for effects. Further, there is some discrepancy between risk perception and state of the research. Some of this may be driven by scientific work with some serious limitations in reporting and methods. This highlights the need for fact-checking of circulating information about microplastics, but also for addressing the discordance between ecotoxicological risk and how risk is framed within the scientific community.

## Introduction

The study of microplastics as a contaminant is a relatively new research area^1^. Adverse effects caused by microplastics have been shown in laboratory exposure studies on marine organisms—usually derived with microplastic concentrations currently above environmental relevance^2,3^. Projected increases of environmental concentrations towards the next century may lead to detection of ecological effects in the environment^4,5^. Little is known about potential risks to human health^6^. So far, exposure has been confirmed by the detection of microplastics in human food sources and in the atmosphere. Microplastics have been found in urban air^7^ and repeatedly in sea salt, seafood and drinks^8–11^. Ingestion through subsequent evacuation was shown for the first time from human stool samples^12^, no research on potential retention, or toxicological or pathological effects exist. Information about inhalation of microplastics is based on studies on factory workers with large exposure to synthetic fibres^7^. In an ecotoxicological context, the risks posed to the environment and organism health are still uncertain and evidence often weak^13,14^. This uncertainty partly derives from disparity between microplastics (types, sizes and characteristics) found in the environment and those used in laboratory studies to assess possible effects^14^. Many potential effects have been extrapolated from macroplastic to microplastic level, especially potential toxicological harm from microplastics’ associated chemicals or adhered pollutants (see review by Rist et al*.*^15^). Further uncertainty relates to the robustness of the scientific methods of individual studies. Inadequate sampling, for example, can challenge the informative value of research concerning quantification and trends of microplastic concentrations, but also comparisons between studies^16^—adding to the difficulties in demonstrating any harm to organisms or ecosystems.

Interest into microplastics research goes beyond the scientific community. People are likely to have heard about microplastics through the media, including print and digital press articles, documentaries and environmental non-profit non-governmental organisations’ (NGOs) social media campaigns^17^. Claims by these stakeholders often deviate greatly from the currently existing scientific knowledge about microplastic pollution^13^. Activism campaigns can successfully influence policy, albeit at times using arguments based on weak of or flawed evidence. In 1995 the successful Greenpeace media campaign against the decommissioning of the Brent Spar oil storage facility was based at least partly on flawed data^18,19^. More recently, the microbeads ban in cosmetics^20^ was based on campaigns supported by weak evidence^21–23^. In addition to such campaigns, scientific ’papers’ or ‘reports’ are being commissioned and published by NGOs and media outlets, without it being clear whether they have had formal independent peer review (see Boyle and Sheets^24^; Santillo et al*.*^25^).

News reports and scientific publications do not always reflect the scientific findings of microplastics research. Völker et al*.*^26^ investigated media article topics from the UK and the US that framed environmental risks. One of the three main uncovered narratives was that microplastics in the human food chain adsorb but also leach harmful chemicals^26^. No distinction had been made between pollutants adhering to particles and chemicals leaching from the internal structure of the particles^26^. Furthermore, “other exposure pathways of these chemicals (e.g., contaminated food) do not play a role as this would change the focus of the storyline” (Völker et al*.* 2019:6^27^). This narrative—humans being the end recipient of microplastics and harmful chemicals—was conveyed in 46% of the articles. A further 14% added potential links between human health and microplastic exposure, linking those occurrences to cancer, hormone disruption and passing the blood/brain or cell barriers^26^. Only 7% of the articles communicated in a factual and neutral manner without speculations or interpretations^26^. Similarly, Hoffmann and Walter^27^ assessed how the German media covered the topic of microplastics. Between 2012 and 2017, only one of 15 articles was of ‘excellent’ journalistic quality based on an assessment tool by the Technical University of Dortmund (medien-doktor.de), with a further four being of ‘good’ journalistic quality^27^. Such reporting issues have also been found in scientific publications. Völker et al*.*^26^ further assessed scientific publications and found that a hypothetical environmental risk (i.e. surrounded by uncertainty and lack of knowledge) was stated in 2/3 of the 464 analysed publications, while an actual risk was reported by almost 1/4. Interestingly, hypothetical risks were reported by effect studies while monitoring studies often stated a risk^26^. Furthermore, stating of risks was often done without providing relevant evidence but rather referring to presence of microplastics in the environment or their ingestion by organisms in addition to saying that the impacts are unknown^26^. Reasons for such framing issues in scientific publications have been attributed to publication bias and disproportionate increase in scientific studies compared to knowledge generation^5,13^.

In the light of misrepresentation of issues relating to microplastics we need to ask ourselves if risk terminology is used appropriately not only by the public but by scientists alike. It seems that even topic-experienced people exhibit a perception bias which may be reflected in poor evaluation of the existing literature, potentially through a flawed understanding of risk. Here we present some preliminary results from a larger global internet survey, quantifying uncertainty in perception for the first time. While most results were informative only for directing our own further research, we believe that the findings presented here will be useful for the scientific community and help explore some of the underlying factors of such misrepresentation and uncertainty. Our research was guided by the following questions: What is the level of concern about microplastics in relation to other environmental issues, what are the reasons for concern about microplastics, how do people perceive the hazardousness of microplastics, and do concern levels and hazardousness perception differ between lay-people and people academically or professionally versed in the topic?

## Results

### Levels of environmental concern

**Figure 1:**
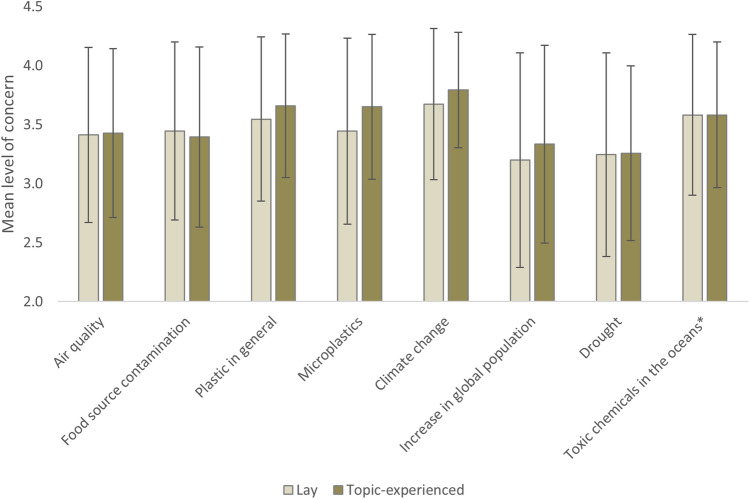
Mean level of concern (± standard deviation) of selected environmental issues of lay respondents (n = 1430) compared to topic-experienced^ respondents (n = 251). Survey results obtained in 2018/2019. *such as pesticides or heavy metals. ^acknowledged their previous or current work on the topic of plastics and/or microplastics as an environmental contaminant or involvement in a research project on this topic. Levels of concern: not concerned (1), slightly concerned (2), moderately concerned (3) and very concerned (4). See Table 1 for differences in rankings between environmental issues per group.

Respondents rated their level of concern for a list of eight environmental issues (mean concern ± 95% CI). Climate change was the most concerning issue for lay respondents and topic-experienced respondents alike. Mean values indicated respondents to be moderately (3) to very (4) concerned on all issues (Fig. 1). Microplastics were ranked fifth by lay respondents (3.4 ± 0.043) and third by experienced respondents (3.7 ± 0.076). Respondents’ median level of concern was ‘very’ for most issues for both groups. The exceptions were global population increase and drought for lay respondents, and drought for topic-experienced respondents, which were all rated as ‘moderate’ concerns. The proportion of lay people who selected ‘don’t know’ ranged 0.5–2.0% for environmental issues, except for microplastics with 8.3%. Similarly, the proportion of topic-experienced people for this selection ranged 0.0–1.2%, except for microplastics with 3.2%. There was a significant difference in concern rankings between groups (WTS (1) = 4.94, p > 0.05) and within-group concern ranking (WTS (7) = 534.72, p < 0.001). Post-hoc exploration of within-group concern ranking differences showed significant differences between most environmental issues (Table 1).

**Table 1:** Post-hoc analysis results: statistical significance between environmental concern rankings per group [(A) lay respondents, (B) topic-experienced respondents] analysed with pairwise comparison Wilcoxon signed rank tests and Bonferroni correction.

	Air quality	Food source contamination	Plastic in general	Microplastics	Climate change	Increase in global population	Drought
**A**							
Food source contamination	1						
Plastic in general	< 0.01	< 0.001					
Microplastics	0.226	< 0.05	1				
Climate change	< 0.001	< 0.001	0.134	< 0.01			
Increase in global population	1	1	< 0.001	< 0.05	< 0.001		
Drought	< 0.05	0.145	< 0.001	< 0.001	< 0.001	1	
Toxic chemicals in the oceans*	< 0.05	< 0.01	1	1	< 0.01	< 0.01	< 0.001
**B**							
Food source contamination	1						
Plastic in general	< 0.001	< 0.001					
Microplastics	< 0.001	< 0.001	1				
Climate change	< 0.001	< 0.001	0.28	1			
Increase in global population	1	1	< 0.001	< 0.001	< 0.001		
Drought	0.07	1	< 0.001	< 0.001	< 0.001	1	
Toxic chemicals in the oceans*	0.08	< 0.01	1	0.24	< 0.001	< 0.05	< 0.001

When asked a second time about their concern, but without the context of other environmental issues, lay respondents’ mean concern about microplastics in the environment stayed at 3.4 ± 0.042; the number of respondents not being concerned fell from 2.9 to 2.0% and respondents expressing ‘don’t know’ from 8.3 to 7.3%. For topic-experienced respondents, when asked again for their level of concern about microplastics without context of other environmental issues fell from 3.7 to 3.6 ± 0.076; number of respondents not being concerned changed from 0.8 to 0.4% and selecting ‘don’t know’ from 3.2 to 2.4%. Asked about changes in concern level over the last year, most respondents’ concern about microplastics had increased in the year prior to the survey (63% of experienced and 59% of lay respondents), with 35% of both groups not having had a change in concern.

### Reasons for concern and perceived hazardousness

**Figure 2:**
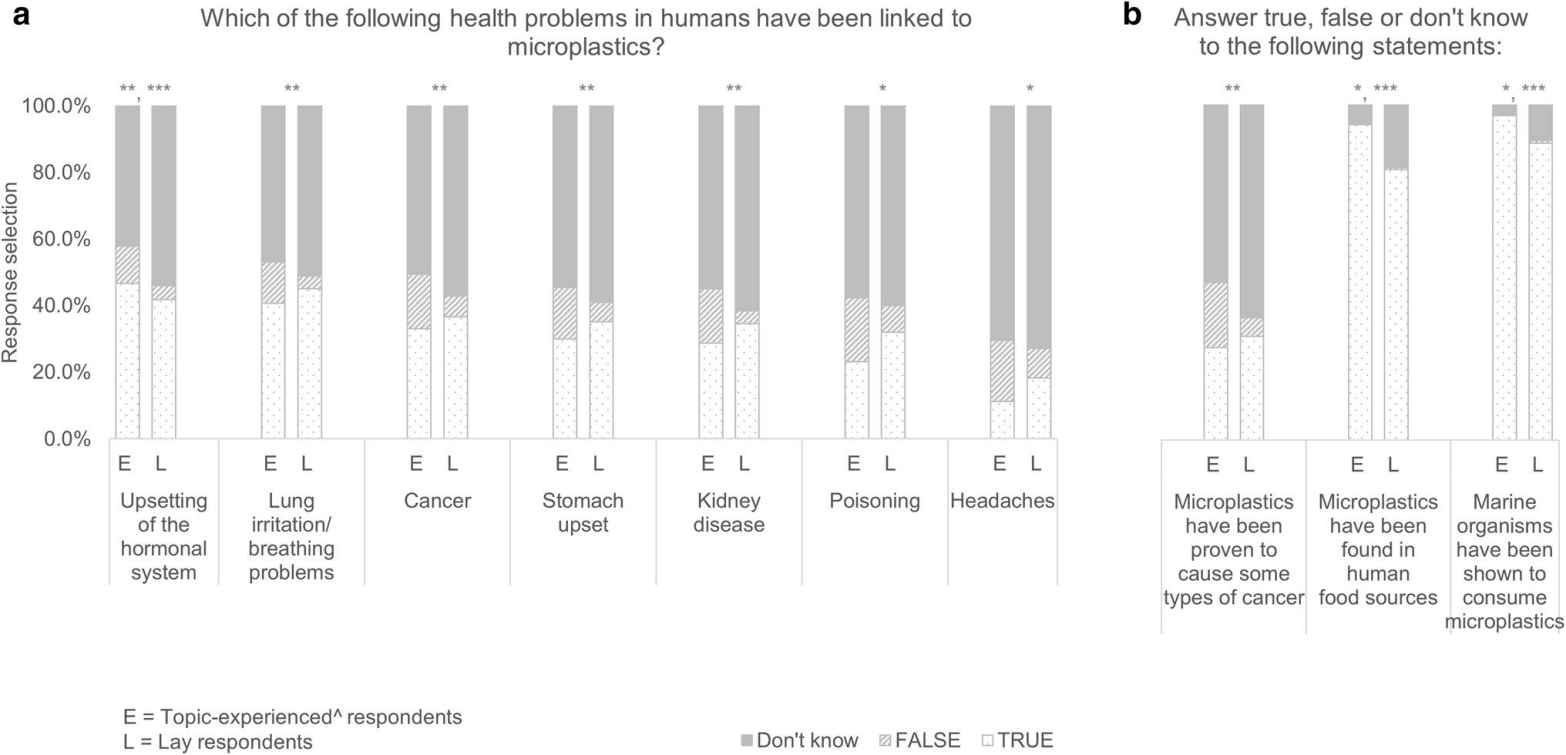
Response selection of lay respondents (n = 1430) and experienced^ respondents (n = 251) to (**a**) ‘Which of the following health problems in humans have been linked to microplastics?’ and (**b**) ‘Answer true, false or don't know to the following statements:’. Statistical test results see Table 2; statistical difference between categorical answers: *true, **false, ***don’t know. Survey results obtained in 2018/2019. ^acknowledged their previous or current work on the topic of plastics and/or microplastics as an environmental contaminant or involvement in a research project on this topic.

Respondents were asked about their reasons for concern about microplastics. ‘Pollution of the marine environment’ was selected most often (93% of experienced and 86% of lay respondents), followed by ‘might contaminate food sources’ (76% and 64%), ‘might enter drinking water sources’ (73% vs 61%), ‘pollution of land’ (55% and 52% respectively) and ‘might be inhaled if suspended in the air’ (38% and 28% respectively). The perceived hazardousness of microplastics was investigated. Respondents were provided with a list of health implications and asked which ones had been linked to microplastics; between 54 and 73% of lay respondents and 41–71% of topic-experienced selected ‘don’t know’, 18–45% of lay and 11–47% of topic-experienced respondents selected ‘true’ and 4–9% of lay and 11–19% of topic-experienced respondents selected ‘false’ (Fig. 2a). Respondents were asked twice about cancer; when asked if microplastics had been linked to cancer (Fig. 2a), 37% of lay and 33% of experienced respondents were affirmative and 57% of lay and 51% of topic-experienced respondents were uncertain. When asked if microplastics had been proven to cause some types of cancer, 31% (lay) and 28% (experienced) confirmed this statement and 64% of lay and 53% of topic-experienced respondents expressed uncertainty (Fig. 2b). Respondent evaluated two additional statements; if microplastics had been found in human food sources and if marine organisms had been shown to consume microplastics (Fig. 2b). Here, uncertainty was low; 11–19% of lay and 3–6% of topic-experienced respondents selected ‘don’t know’. Less than 1% of all respondents selected ‘false’ (Fig. 2b). For all health implications and other statements, there was a significant between-group difference (Table 2). Post hoc testing revealed that, for the human health implication questions, this was due to significantly more experienced respondents selecting ‘false’ to all responses, but also that significantly more lay respondents selected ‘don’t know’ to upsetting the hormonal system (Table 2).

**Table 2:** Results of between group (lay/experienced) χ^2^ test and subsequent posthoc (residual chi-square) test to questions posed in Fig. 1. Bonferroni-adjusted p-value of 0.004.

Sub-question according to Fig. 1	χ^2^	df	p	Significant difference according to posthoc test in response selection (between ‘true’, ‘false’, ‘don't know’)	Notes
Upsetting of the hormonal system	25.98	2	< 0.000001	‘False’ (p < 0.0001); ‘don't know’ (p < 0.004)	
Lung irritation/breathing problems	31.83	2	< 0.000001	‘False’ (p < 0.000001)	
Cancer	30.61	2	< 0.000001	‘False’ (p < 0.000001)	
Stomach upset	29.02	2	< 0.000001	‘False’ (p < 0.000001)	
Kidney disease	62.04	2	< 0.000001	‘False’ (p < 0.000001)	
Poisoning	32.61	2	< 0.000001	‘False’ (p < 0.000001)	
Headaches	24.75	2	< 0.000001	‘False’ (p < 0.000001)	
Microplastics have been proven to cause some types of cancer	60.65	2	< 0.000001	‘False’ (p < 0.000001)	
Microplastics have been found in human food sources	26.66	2	< 0.000001	‘True’ (p < 0.00001); ‘don't know’ (p < 0.00001)	Yates correction
Marine organisms have been shown to consume microplastics	16.05	2	0.000001	‘True’ (p < 0.001); ‘don't know’ (p < 0.001)	Yates correction

### Response consistency (data quality)

On two instances respondents were asked the same question again (concern about microplastics, microplastics and cancer). For concern about microplastics, 84.9% topic-experienced respondents provided the same answer both times, 13.5% changed up or down one level of the Likert scale, 0.8% changed two levels on the scale. 0.8% (2 respondents) selected ‘don’t know’ for one question and either ‘very’ or ‘moderately concerned’ for the other. When asked about cancer, respondents were first asked if cancer in humans had been linked to microplastics, and later in the survey if microplastics had been proven to cause some types of cancer. 78.5% of topic-experienced respondents provided the same answer both times, 2.4% gave opposing answers (half of which responded that proof existed but no link), 19.1% chose ‘don’t know’ for one and a different answer (true/false) for the other question. Lay respondents’ consistency was as follows; 74.8% did not change their concern about microplastics between questions, 19.2% changed up or down one level of the Likert scale, 2.8% changed two levels and 0.1% said they were very concerned one time, and not concerned the other. 4.5% of lay respondents chose ‘don’t know’ for one and a response between not and very concerned for the other question. When asked about microplastics and cancer, 83.4% of lay respondents did not change their responses between questions, 1.2% gave opposing answers (half of which responded that proof existed but no link) and 15.4% chose ‘don’t know’ for one and a different answer (true/false) for the other question—mainly ‘true’ (11.2%).

The ‘don’t know’ responses regarding cancer were broken down further: 8.4% of lay people who stated uncertainty to microplastics having proven to cause cancer were affirmative that a link between human cancers and microplastics existed, 2.5% were not. Of lay respondents uncertain if microplastics had been linked to human cancers, 2.8% were affirmative about existing proof that microplastics cause some types of cancer, 1.7% were not. Of topic-experienced people, 8.0% who stated uncertainty to microplastics having proven to cause cancer were affirmative that a link between human cancers and microplastics existed, 2.4% were not. Of the topic-experienced uncertain if microplastics had been linked to human cancers, 2.8% were affirmative about existing proof that microplastics cause some types of cancers 6.0% were not.

## Discussion

Levels of concern for environmental issues expressed in this survey are comparable to previous studies. Climate change or marine/coastal pollution are often stated as the most important general environmental and marine issues^28–31^. Interestingly, topic-experienced respondents expressed a greater concern for microplastics than lay respondents. Previously, it was found that scientists usually rate risks from their own area of expertise as lower than the general public^32,33^. For example, lay people perceive ecosystem impacts through climate change as slightly more severe, but trust in experts regarding understanding the risk, and potential control of the risk^34,35^. Further research is needed to elucidate lay people’s trust in experts on this matter. This increased concern compared to lay respondents is in disagreement with other experts in their field (e.g. see Koelmans et al*.*^13^). Reasons for concern about microplastics are in line with previous work. The latest Eurobarometer reported that 80–90% of European respondents worry about the environmental impact of microplastics^36^. EC^31^ reported in 2017 that respondents are more worried about the impact of plastics on the environment compared to their health (87% vs 74%) and in 2019, 48% of Europeans had heard of microplastics being found in food sources, but only 21% were concerned about it^37^.

People seem more uncertain if they should be concerned about microplastics compared to other environmental issues. Also, widespread uncertainty exists surrounding knowledge about microplastics in lay people and topic-experienced respondents alike. Even when asking vaguely (i.e. health problems in humans having been linked to microplastics) rather than asking if proof existed, over half of the respondents stated that they did not know. Uncertainty was much lower for knowledge about marine organisms consuming microplastics and the existence of microplastics in human food sources. Those two pieces of information are well-researched and most often covered by the media^26,38,39^.

To a lesser degree compared to respondents expressing uncertainty about existing evidence or links to a suite of effects, almost 1/3 of people—including topic-experienced ones—were affirmative about existing evidence or links. This is despite evidence gathered to date suggesting that no proven health effects for humans from microplastics exist^4–6,13–15^. The statement that was affirmed most among experienced and second-most among lay respondents was the link between microplastics and the upsetting of the hormonal system. Völker et al*.*^26^ found that “92.8% of media articles imply that risk of microplastics exist and harmful consequences are highly probable” (p.7)^40^. However, in this specific case the evidence provided to date suggests that the chemicals related to plastics in microplastics exert a negligible effect on human health despite commonly being stated in the press, and also in the scientific literature^5,15,26,41^. There is an ongoing debate about the certainty science can put around this and other risks to date^23,42–45^. Indeed, some chemicals associated with plastic production (e.g. phthalates) are suspected to affect the human endocrine system^46^. Handling macroplastic items, such as wearing PVC gloves, mouthing soft plastic toys or food getting into contact with plastic food packaging increases measurable quantities of phthalates, for example, but the half-life in the human body is short^47^. Mechanisms and effects of microplastics are often conjectured from our vast use of much larger items^15^. Humans do ingest microplastics—an umbrella term for a suite of different polymers with a range of additives likely exhibiting different absorption, desorption and leaking characteristics, but their mass and surface area are likely to be well below that of handled macroplastics. Natural particles, food and water are considered the main route of exposure for such chemicals^41,48^. This is not to say that microplastics do not pose a hypothetical risk, but that the evidence—at current microplastic concentrations—does not support most of the statements we put forward in our survey. Extensive work is still needed to adequately assess actual risks microplastics may pose, and toxicology in general is often uncertain.

Nonetheless, our results show that there is discord between how people perceive microplastics and what we actually know about them. An entire field of research is devoted to how people perceive risks. Risk perception is based on “people's beliefs, attitudes, judgements and feelings, as well as the wider cultural and social dispositions they adopt towards threats to things that we value” (Pidgeon 1998:5^49^) as well historical context of the issue in question^50,51^. People’s perception of a potential hazard being seen as a risk may also be based on characteristics of such hazard that experts usually do not use, such as catastrophic potential, threats to future generations, judged controllability and dread; the degree of scientific disagreement may also feed into people’s idea of risk^49,52^. This can be seen in people’s perception of air travel being riskier than car travel despite contrary evidence in general, but especially after memorable events such as terrorist attacks^53,54^. Further influencing factors are: if something is believed not to be natural, when trust in regulatory bodies is lacking, uncertainty exists and the type of emotional response that can be triggered^55^. These deep-rooted psychological mechanisms of risk perception are influenced by ‘risk signals’^52^, i.e. “occurrences that suggest to the public that the risk is more serious or difficult to manage than had been previously assumed” (Kasperson and Kasperson 2005:10^56^). Many of these above-mentioned factors and mechanisms may affect people’s perception of microplastics and regular media coverage and ever-increasing research output may act as such signal.

Our work suggests that risk perception of topic-experienced people is not in line with the state of knowledge of the research area, indicating personal bias potentially causing misconceptions in the field of microplastic research. High levels of misconceptions in this research area have been suggested previously^13,15,26^, but had not been quantified to date. Völker et al*.*^26^ found risk framing of microplastics to be common in the scientific literature, about a quarter of studies present microplastics as an environmental risk. This number increases to almost 1/3 of studies in the area of environmental monitoring^26^. Such studies are concerned with establishing concentrations of microplastics in the environment and therefore do not directly assess potential risks of this contaminant—only the presence and quantity of the hazard. Koelmans et al*.*^13^ suggest that pressures to compete in publishing and obtaining of funding as reasons for microplastics risk framing. This may well be true; however, it may simply be that people versed in (micro-)plastic research are driven by their risk perception, rather than the state of knowledge, therefore introducing their personal bias into the field. This is supported by Völker et al*.*^26^ who found that authors of scientific studies may not use the concept of risk as (eco)toxicologists would do. It may be that authors of said studies understand microplastics’ potential hazardousness as the same as them posing an actual risk to human health based on classical risk assessments.

The most stated health effect among lay and second-most among experienced respondents was that respiratory problems are linked to microplastics. Interestingly, more people were affirmative about this than there were respondents concerned about microplastics possibly being inhaled if suspended in the air. This is a new avenue of research and not much was known when the survey was conducted. The potential of inhalation of microplastics exists, but harm has only been assessed for factory workers exposed to high levels of synthetic fibres (see reviews by Wright and Kelly^6^; Gasperi et al*.*^7^). Up until July 2018, less than 1% of media articles were dedicated to microplastics in the air^26^. It is, therefore, interesting that such a large proportion of respondents were affirmative about a link between health effects from breathing microplastics, while their concern was relatively low, and begs the question were people obtained this insight. A great proportion of people obtain their environmental information via the internet these days^29,31^. Perceptions like the ones we uncovered about microplastics may have come into existence and publicly manifested, during a time that has seen a shift in social interactions, i.e. the rise of user-generated content online. This is amid rising concern over lack of fact checking on social media and the rise of ‘Fake News’. In order to evaluate sources of claims relating to microplastics, outputs by a range of entities that communicate to people, be it mass or social media, internet, government authorities or non-government organisations and other public agencies would need to be assessed. A limited number of conventional news outlets and scientific publications have been scrutinised for their quality of environmental journalism and content^26,27^, but quality and covered topics of other sources of environmental information are yet to be evaluated. To advance the field of microplastics research, it is essential that the quality of microplastics journalism and general distribution of knowledge become of better quality. This must also include scientific publications.

There are limitations to our study. One of these is the focusing effect, where questioning people about environmental issues brings those issues to people’s minds^57,58^. However, we purposefully provided respondents with a list of different issues, which should help to rank microplastics amongst other environmental concerns. When it came to perceived hazards, the relatively large proportion of indeterminate responses suggests that this effect might not be of concern. Secondly, using the wording ‘problems linked to microplastics’ when enquiring about human health effects through microplastics was not ideal. However, respondents did seem to understand this more like if evidence for such effects existed, as the detailed analysis of one of the repeatedly asked questions has shown. Further, disseminating a study online likely converges responses from areas of higher internet and social media penetration. Inherent perils of self-selection exist with online surveys^59^. Through self-selection, our study group is likely to be biased towards respondents interested in the topic^60^. Hartley et al*.*^61^, using a similar approach to ours, obtained respondents with comparable demographics. Potts et al*.*^30^, using a random sampling strategy on the other hand, also reported relatively high levels of university-educated participants. Also, since we expected elevated levels of postgraduate-educated respondents in the topic-experienced group, it is advantageous that demographics between groups are not too dissimilar. Moreover, 25% of experienced and 16% of lay respondents stated to be vegan, vegetarian or not eating seafood for environmental reasons. This suggests a relatively high level of environmental consciousness amongst respondents. Reliable global statistics are scarce, but veganism and vegetarianism in Germany, for example, was 4% in 2016^62^, while 10% of our German participants stated to be vegan or vegetarian. In addition, even in studies without self-selection, is has been found that environmental protection is personally important to people^31^. Responses were relatively similar between different demographic groups (see Supplementary Information), suggesting robustness of our results.

## Conclusion

There is a great deal of uncertainty surrounding the knowledge about microplastics, but also some discrepancy between risk perception and the state of research. Previously suggested by others and confirmed by our results, there seems to be a personal perception bias in people with professional expertise on the topic. A consequence of this bias is likely the discordance between ecotoxicological risk and how risk is framed in the scientific literature around the effects of microplastics. To fully understand this, it is important to qualitatively scrutinise information about microplastics disseminated by the media and through scientific literature alike.

## Data and methods

A survey was designed in English and translated to Spanish, German, Italian, French, Polish, Greek, Croatian, Japanese, Thai, Indonesian, Malay, Portuguese, Chinese and Arabic. Back-translation via Google Translate was performed as an additional check. Survey data were collected via SurveyMonkey (www.surveymonkey.co.uk) and the link distributed via social media (Twitter, Facebook and LinkedIn), and messaged directly to the private and professional networks of the authors. Data collection took place between September 2018 and May 2019. All participants were 18 years or older, informed consent was obtained prior to the start of the survey. The questionnaire was attempted by 2084 people. After data cleansing the final sample consisted of 1681 responses. Questions were closed questions. Responses either had to be chosen from a list of options (depending on the question, either single or multiple answers could be selected), required a response on a Likert scale (‘not, slightly, moderately or very concerned’) or ‘true/false’, both with the addition of ‘Don’t know’^63^. Questions related to awareness, concerns and perceived hazards of microplastics. Alongside the survey questions (see Supplementary Section S.1 in Supplementary Information), the following demographic information was collected. While country of residence was initially collected, this is not taken into account here due to unbalanced numbers of responses (available at https://doi.org/10.5258/SOTON/D1379).

- Topic experience. The large response rate of topic-experienced people allowed for in-depth analysis between lay and experienced respondents. This was measured by asking respondents “Do you currently work or have you ever worked on the topic of plastics as an environmental contaminant (e.g. microplastics, plastic pollution, marine debris, effects of microplastics on organisms, etc.)? Or/and Are you or have you been involved in a research project on this topic (e.g. BSc or MSc final year project, PhD project etc.)?” as the last question of the survey. Respondents answering ‘no’ are defined as lay respondents (n = 1430) and as topic-experienced when answered ‘yes’ (n = 251).
- 65.3% of lay and 65.7% of topic-experienced respondents were female, 33.5% and 33.1% respectively male, the remainder of respondents opted for ‘prefer not to say’.
- Age: 14.3% of lay and 21.5% of experienced respondents were 18–24 years, 64.2% and 65.7% respectively were 25–44 years, 17.2% and 10.8% were 45–64 year, 3.6% and 1.6% were 65 years and above, while 0.7% of lay and 0.4% of experienced respondents did not want to disclose this information.
- Highest level of education completed: 0.3% of lay and 0.4% of experienced respondents did not have any qualifications, 13.4% and 7.6% respectively had completed secondary school or equivalent, 36.4% and 28.7% possessed an undergraduate degree or had completed trade/technical/vocational training, 49.1% and 63.3% respectively possessed a postgraduate degree, while 0.8% of lay respondents did not want to disclose this information.

Statistical analyses were conducted with RStudio 1.0.153^64^. Concern ratings were presented as means ± 95% confidence interval (CI; and standard deviation). Between group and within group differences (environmental issues of concern) were set to be analysed using a mixed-design ANOVA. This is a similar approach to Hartley et al*.*^61^ who used a series of one-way repeated measures ANOVAs, but accommodates a between-subjects variable. Assumptions were not met for normality (Anderson–Darling test), homogeneity of variances (Levene’s test) and sphericity (Mauchly’s sphericity test). The ‘f1.ld.f1’ function (R software package nparLD^65^) was used as a non-parametric equivalent and its Wald-type statistic (WTS) reported^66^. ‘Don’t know’ answers were coded as ‘0’. Post-hoc testing was performed with a pairwise comparison Wilcoxon signed rank test and Bonferroni correction^67^. Between-group differences for statements relating to reasons for concern about microplastics were assessed with Pearson’s chi-squared tests to test for associations (Yates Correction when < 5 responses in a category) between answers comparing frequency counts of each possible answer (true, false and don’t know)^68^. Post hoc testing was performed with the residuals of the Pearson’s χ^2^ using the package ‘chisq.posthoc.test’^69^. A Bonferroni correction was applied for multiple χ^2^ tests (12 tests for questions), reducing the α-value to 0.004. To assess response consistency, two questions were asked twice: concern level regarding microplastics was questioned twice (once in context of other environmental issues, once on its own) and with reverse scales; and a statement regarding cancer. A percentage distribution result analysis by demographics can be found in the Supplementary Section S.2.

### Ethical approval

Ethical approval for this research was granted by Ethics and Research Governance Online (ERGO REF41938) at the University of Southampton in compliance with the UK Concordat to Support Research Integrity. All methods were carried out in accordance with highest current international standards of ethical best practice.

## Supplementary Information

Supplementary Information.

## Data Availability

All data supporting this study are openly available from the University of Southampton repository at https://doi.org/10.5258/SOTON/D1379.
