# Supplementary material for: Uncertainty about the risks associated with microplastics among lay and topic-experienced respondents
Source: Sci Rep. 2021 Mar 30;11:7155. doi: 10.1038/s41598-021-86569-5 (PMC8009892; doi:10.1038/s41598-021-86569-5)
Supplement: Supplementary file 1 — Supplementary Information. [file 41598_2021_86569_MOESM1_ESM.pdf]

Supplementary information for

Uncertainty about the risks associated with microplastics among lay and topic-experienced respondents

Thiele, Christina J.* and Hudson, Malcolm D.

Centre for Environmental Science, Faculty of Environment and Life Sciences, University of Southampton, University Road, Southampton SO17 1BJ, UK

*Email address corresponding author: c.j.thiele@soton.ac.uk and mdh@soton.ac.uk

S.1 Questions from the entire survey that were used for this publication

2. Rate your level of concern for the following environmental issues:
(Please rate every option given)

	Not concerned	Slightly concerned	Moderately concerned	Very concerned	Don't know
Air Quality	○	○	○	○	○
Contamination of food sources	○	○	○	○	○
Plastics in general	○	○	○	○	○
Microplastics	○	○	○	○	○
Climate change	○	○	○	○	○
Increase in global population	○	○	○	○	○
Drought	○	○	○	○	○
Toxic chemicals such as pesticides or heavy metals in the oceans	○	○	○	○	○

4. How concerned are you about microplastics in the environment?
(Please select only one answer)

- ◯ Very concerned
- ◯ Moderately concerned
- ◯ Slightly concerned
- ◯ Not concerned
- ◯ Don't know

5. Why are you concerned about microplastics in the environment?
(Please select at least one answer)

- ☐ Pollution of the marine ecosystem
- ☐ Pollution of land
- ☐ Might enter drinking water sources
- ☐ Might contaminate food sources
- ☐ Might be inhaled if suspended in the air
- ☐ Other (please specify)
- ☐ Don't know

Other (please specify)

6. Are you more concerned now than you were 1 year ago about microplastics in the environment?
(Please select only one answer)

- ◯ More concerned
- ◯ Less concerned
- ◯ No change
- ◯ Don't know

10. Which of the following health problems in humans have been linked to microplastics?

(Please select only one answer per statement)

	True	False	Don't know
Cancer	○	○	○
Kidney disease	○	○	○
Lung irritation/ breathing problems	○	○	○
Stomach upset	○	○	○
Poisoning	○	○	○
Headaches	○	○	○
Upsetting of the hormonal system (which is regulating bodily functions such as growth, sexual development and metabolism)	○	○	○

11. Answer true, false or don't know to the following statements:

(Please only select one response for each statement)

	True	False	Don't know
Marine organisms have been shown to consume microplastics	○	○	○
Microplastics have been found in human food sources	○	○	○
Microplastics have been proven to cause some types of cancer	○	○	○

15. How often do you eat seafood (shellfish and fish)?

(Please select only one answer)

- ○ Once a month
- ○ 2 to 4 times a month (e.g. once a week or less)
- ○ 5 to 8 times a month (e.g. once to twice a week)
- ○ 9 to 10 times a month (.g. twice a week, occasionally three times a week)
- ○ 11 or more times a month
- ○ I do not eat seafood even though I do eat meat, chicken, etc. (e.g. I do not like seafood, I am allergic to seafood, etc)
- ○ I am vegan/vegetarian and therefore do not eat seafood
- ○ I do not eat seafood for environmental reasons even though I do eat other animal products (i.e I am NOT a vegan or vegetarian)

18. In what country do you live?

19. How long have you lived in that country?
(Please select only one answer)

- (Almost) all my life
- Less than 2 years
- 2 years or longer

20. What is your gender?
(Please select only one answer)

- Female
- Prefer not to say
- Male

21. What is your highest level of education?
(Please select only one answer)

- No qualifications
- Master degree
- Secondary school or equivalent
- Doctorate degree
- College (16-18 years) or equivalent
- Prefer not to say
- Trade/technical/vocational training
- Other (please specify)
- Undergraduate degree or equivalent

Other (please specify)

22. How old are you?
(Please select only one answer)

- 18-24 years
- 65 years or above
- 25-44 years
- Prefer not to say
- 45-64 years

23. Do you currently work or have you ever worked on the topic of plastics as an environmental contaminant (e.g. microplastics, plastic pollution, marine debris, effects of microplastics on organisms, etc.)?
Or/and
Are you or have you been involved in a research project on this topic (e.g. BSc or MSc final year project, PhD project etc.)?

- Yes
- No

S.2 Percentage distribution of results by the demographics of sex, education and age

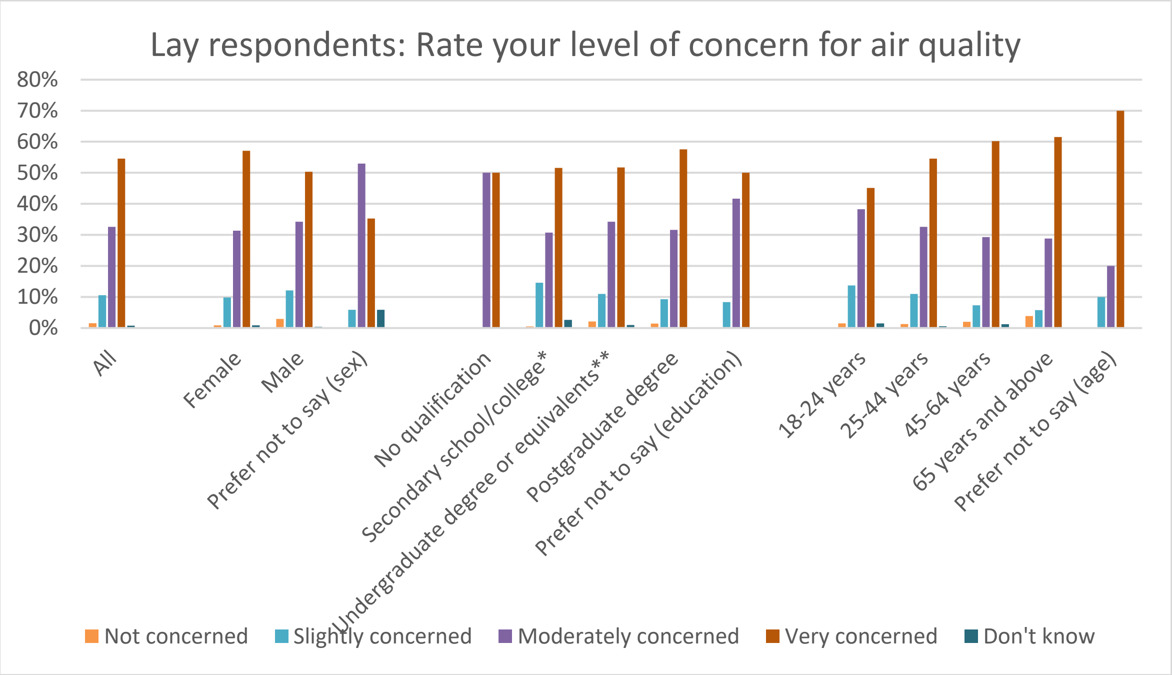

Figure S1 – Percentage distribution of responses of lay respondents by demographics (sex, education and age) for air quality, * (16-18 years) and equivalents, ** Including trade/technical/vocational training.

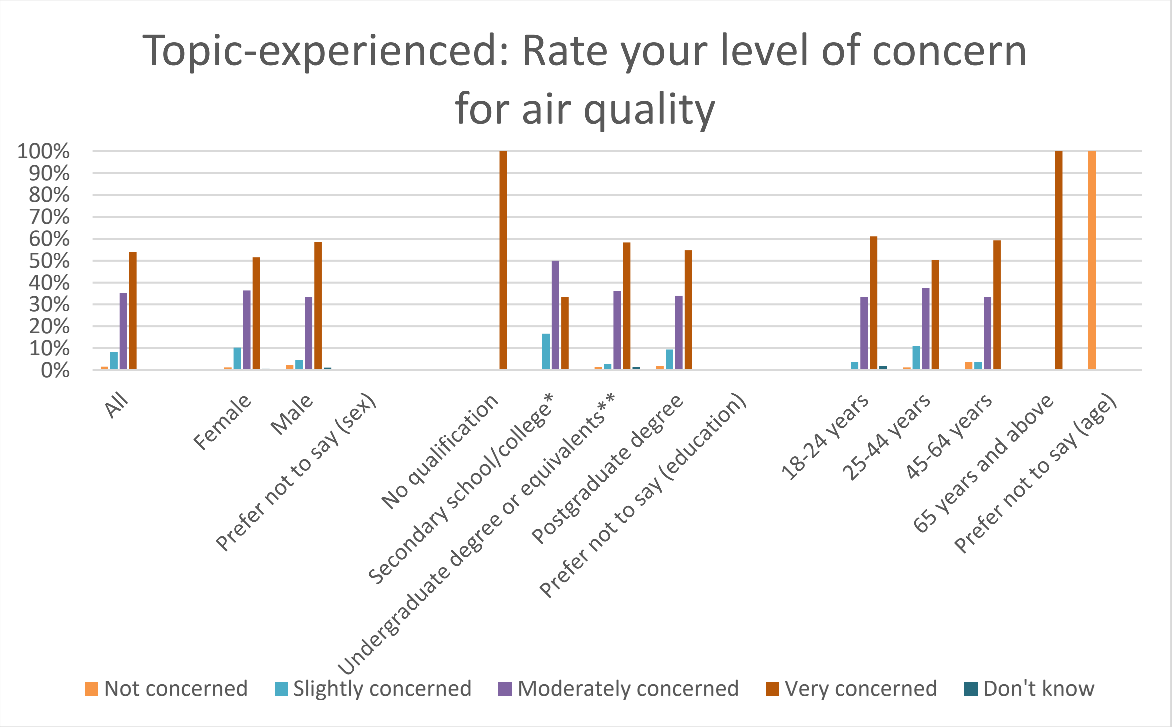

Figure S2 - Percentage distribution of responses of topic-experienced respondents by demographics (sex, education and age) for air quality, * (16-18 years) and equivalents, ** Including trade/technical/vocational training.

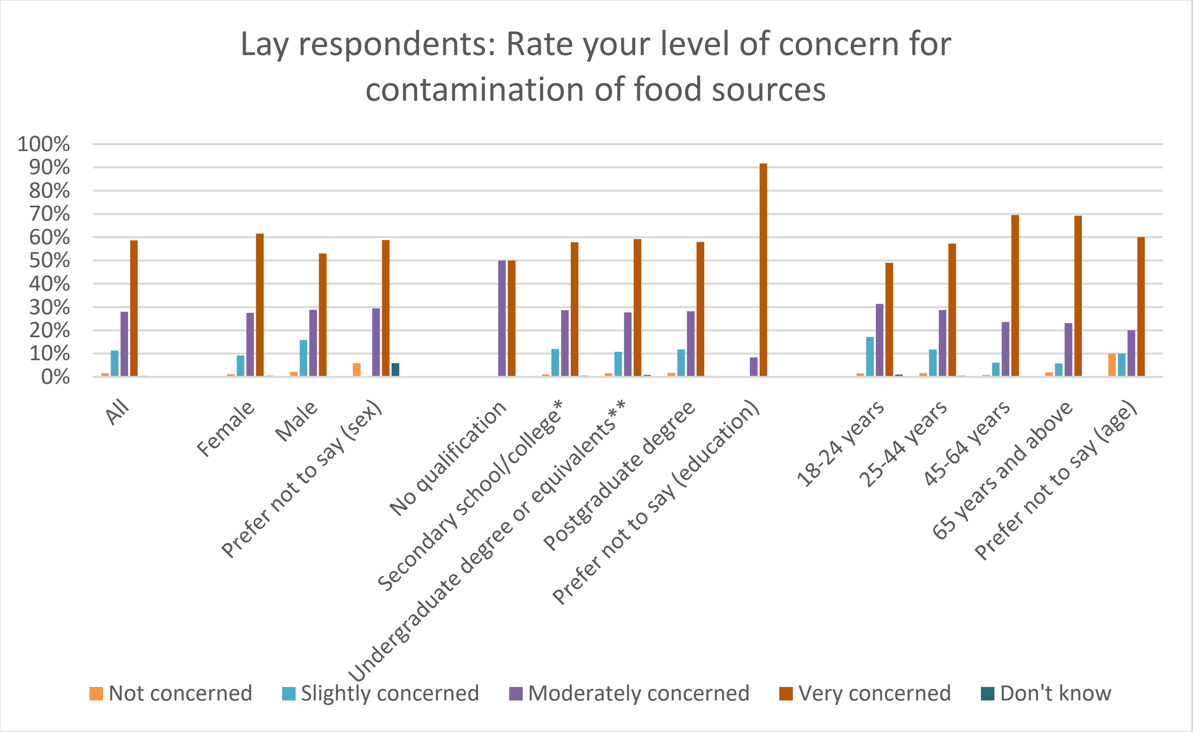

Figure S3 - Percentage distribution of responses of lay respondents by demographics (sex, education and age) for contamination of food sources,* (16-18 years) and equivalents, ** Including trade/technical/vocational training.

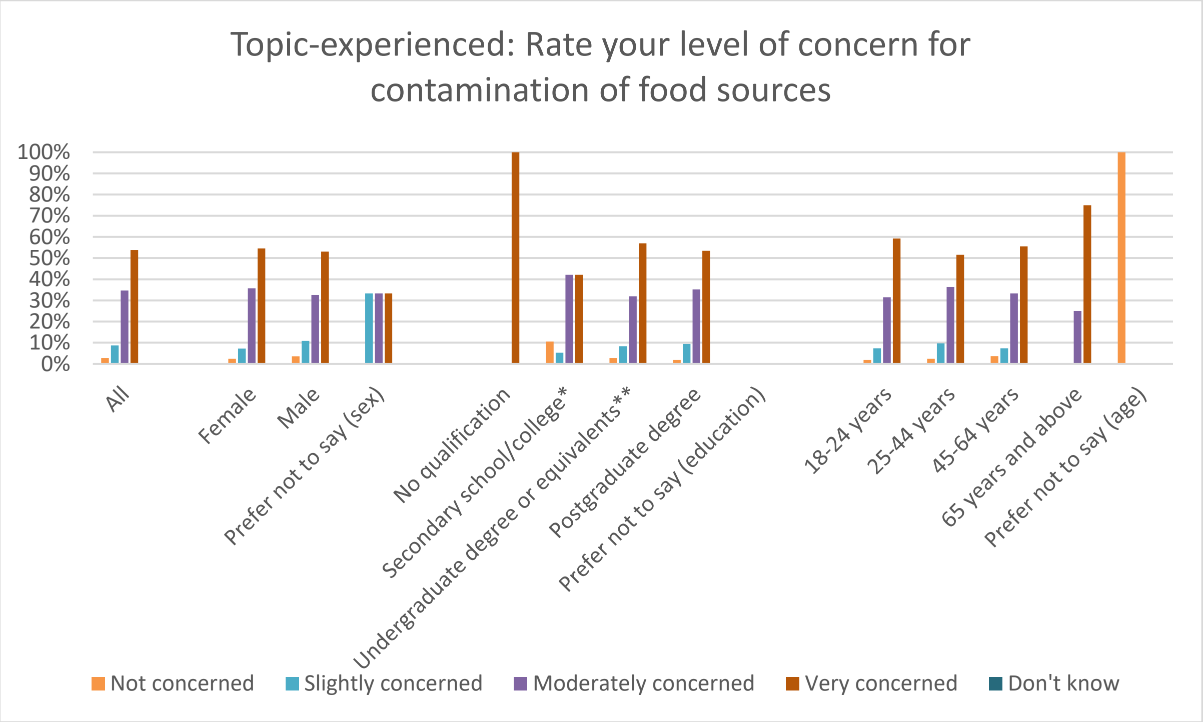

Figure S4 - Percentage distribution of responses of topic-experienced respondents by demographics (sex, education and age) for contamination of food sources,* (16-18 years) and equivalents, ** Including trade/technical/vocational training.

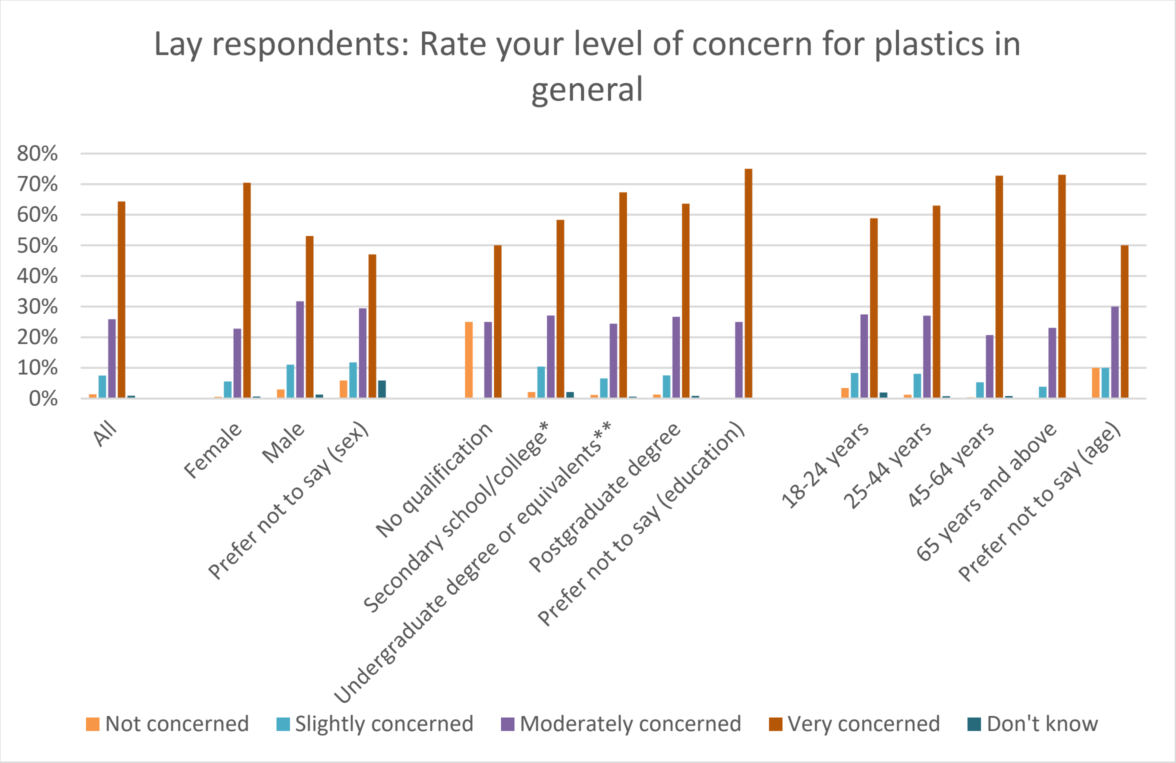

Figure S5 - Percentage distribution of responses of lay respondents by demographics (sex, education and age) for plastics in general,* (16-18 years) and equivalents, ** Including trade/technical/vocational training.

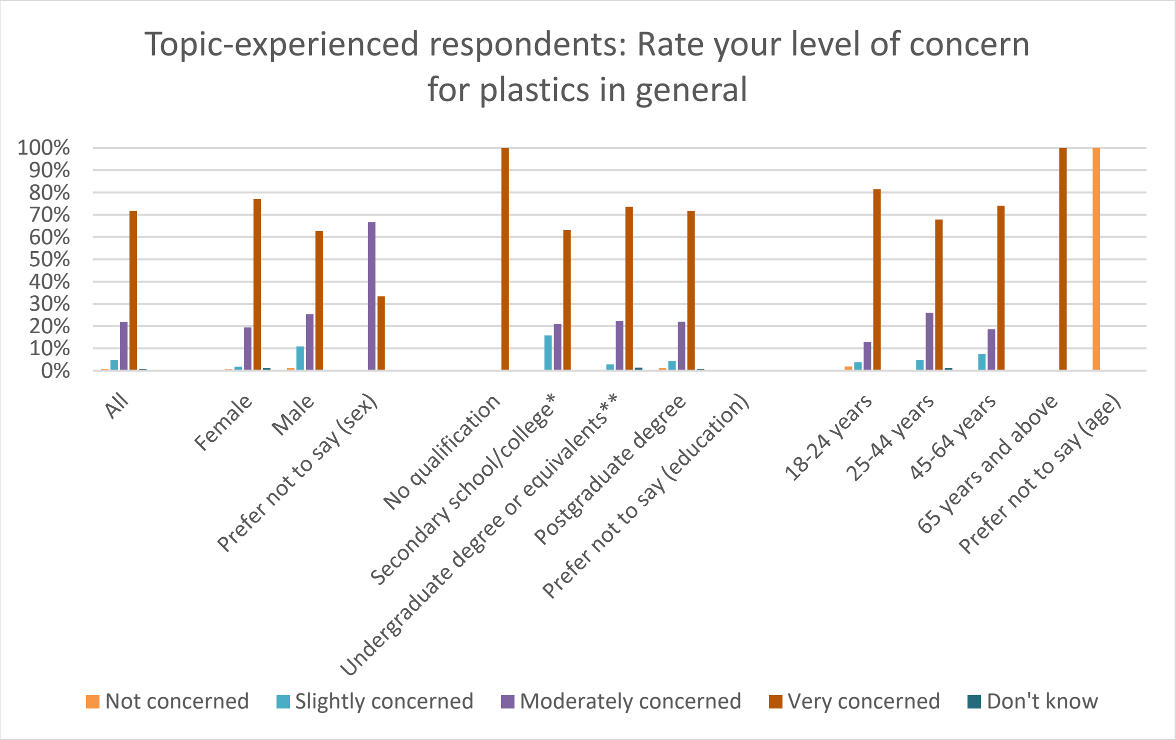

Figure S6 - Percentage distribution of responses of topic-experienced respondents by demographics (sex, education and age) for plastics in general,* (16-18 years) and equivalents, ** Including trade/technical/vocational training.

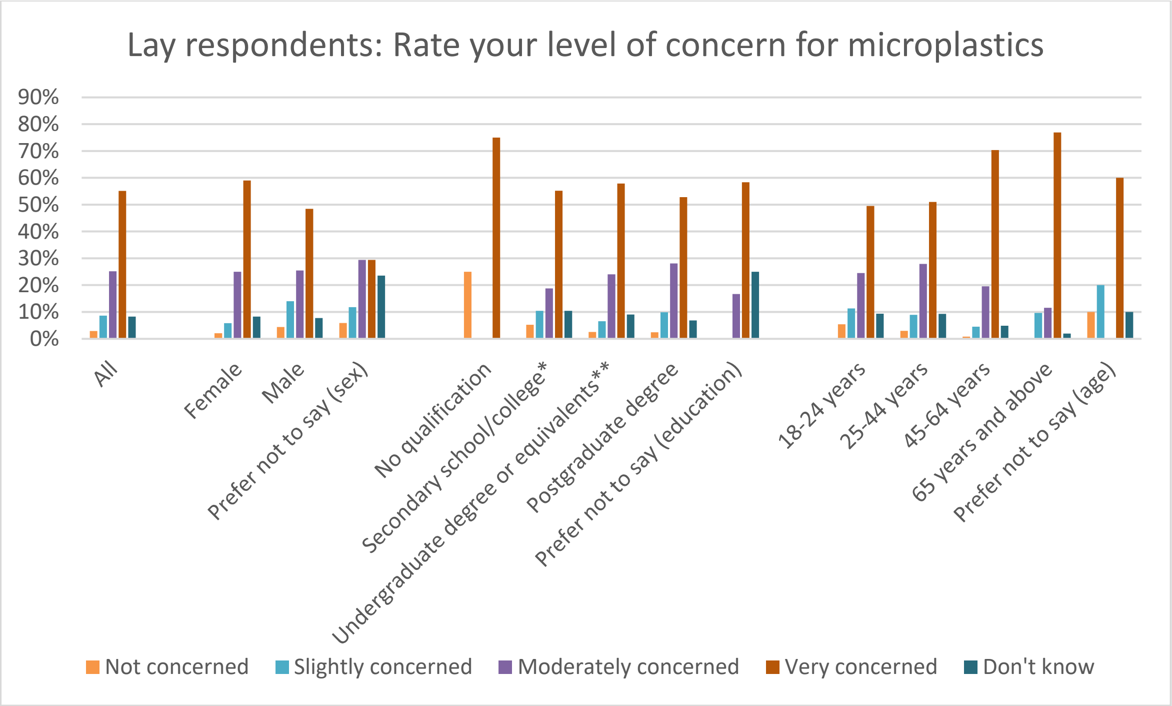

Figure S7 - Percentage distribution of responses of lay respondents by demographics (sex, education and age) for microplastics ,* (16-18 years) and equivalents, ** Including trade/technical/vocational training.

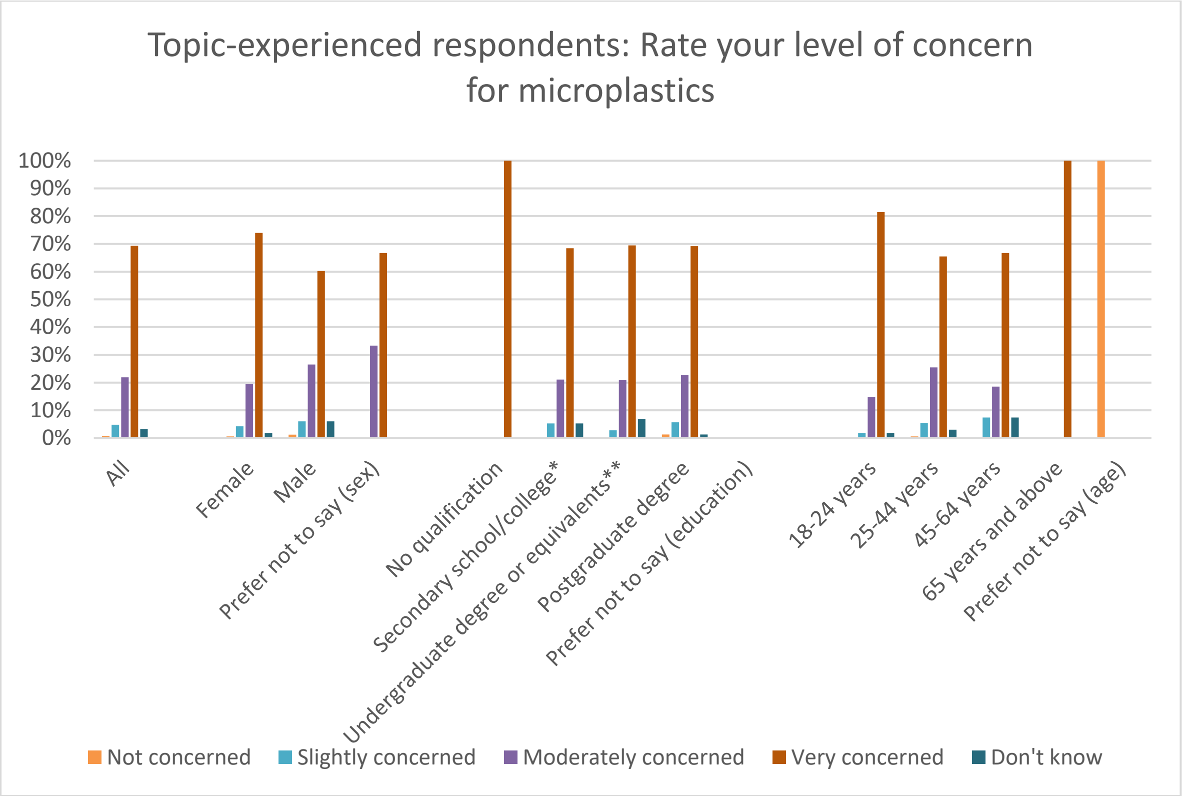

Figure S8 - Percentage distribution of responses of topic-experienced respondents by demographics (sex, education and age) for microplastics,* (16-18 years) and equivalents, ** Including trade/technical/vocational training.

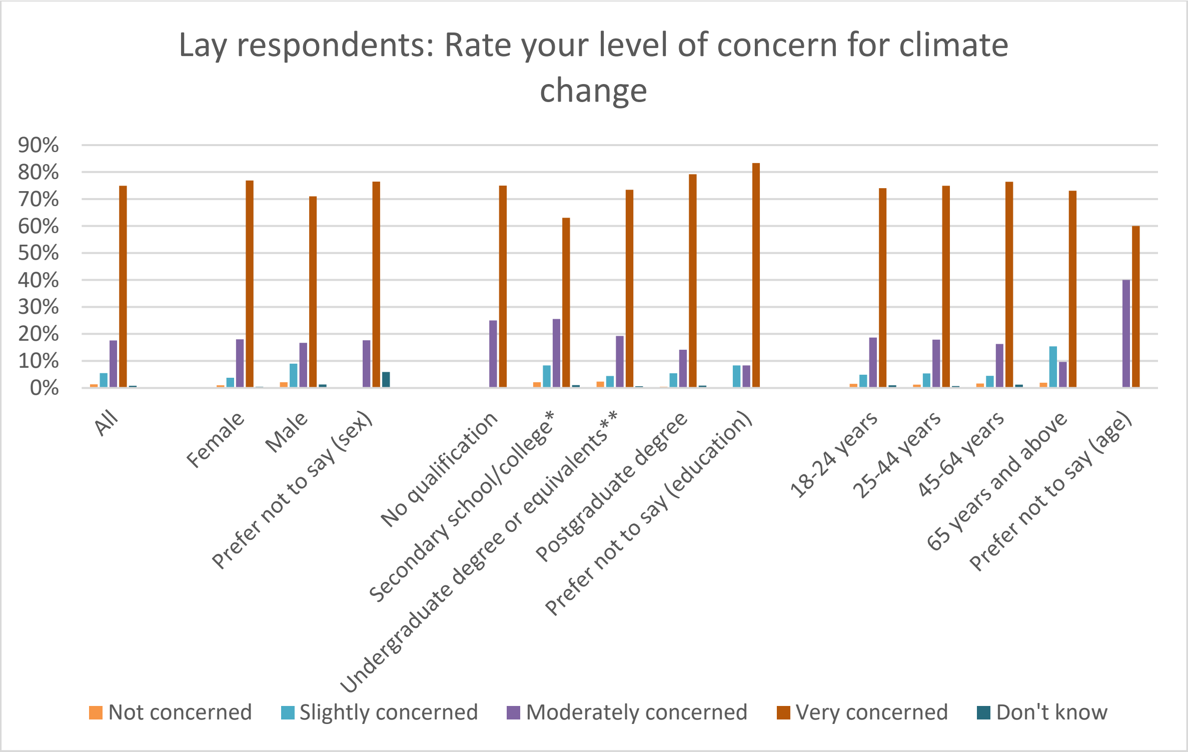

Figure S9 - Percentage distribution of responses of lay respondents by demographics (sex, education and age) for climate change,* (16-18 years) and equivalents, ** Including trade/technical/vocational training.

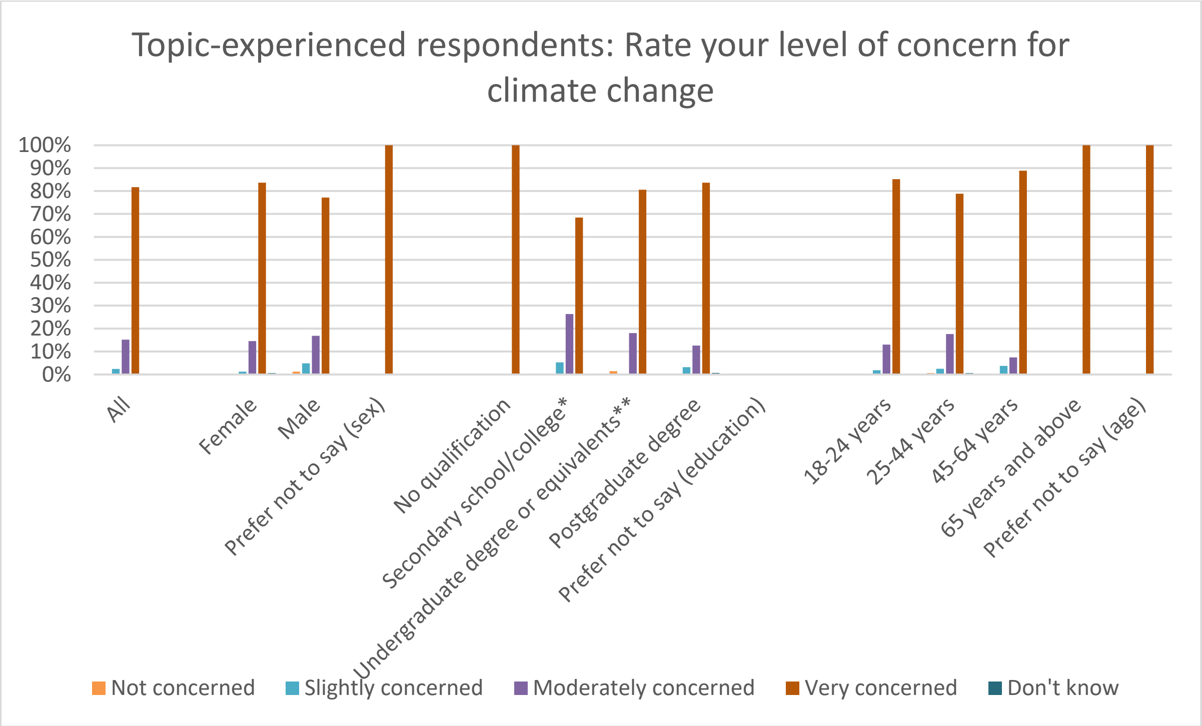

Figure S10 - Percentage distribution of responses of topic-experienced respondents by demographics (sex, education and age) for climate change,* (16-18 years) and equivalents, ** Including trade/technical/vocational training.

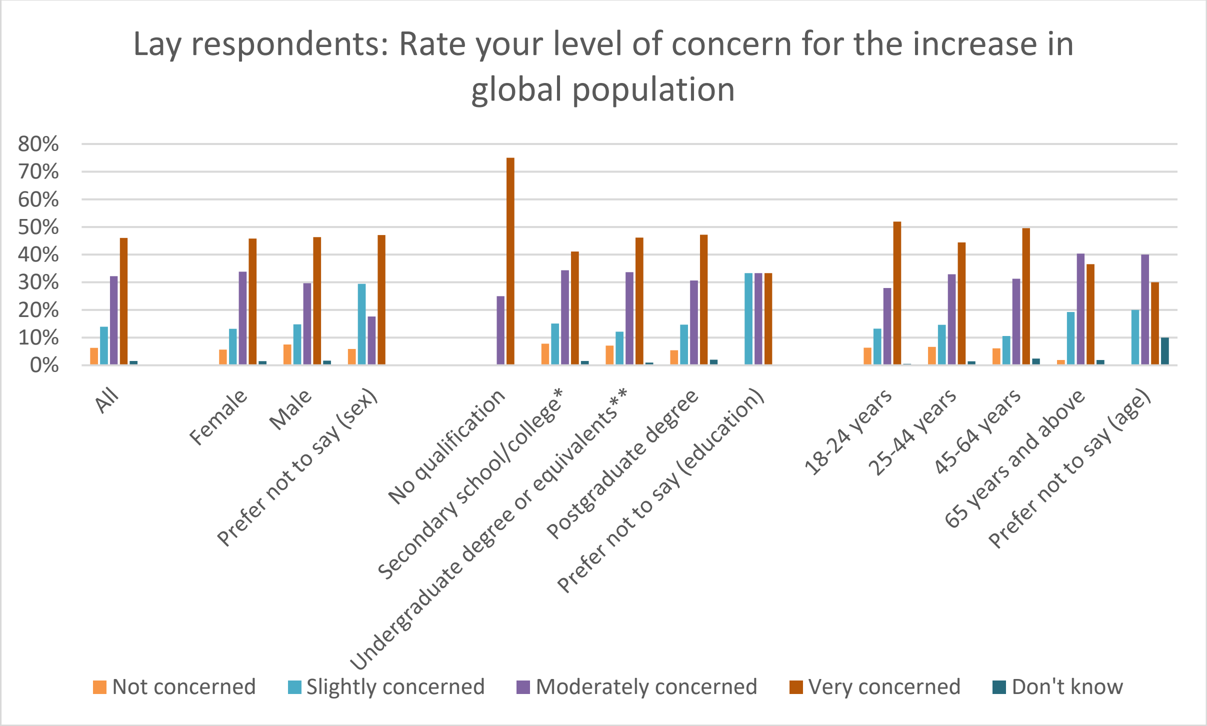

Figure S11 - Percentage distribution of responses of lay respondents by demographics (sex, education and age) for increase in global population,* (16-18 years) and equivalents, ** Including trade/technical/vocational training.

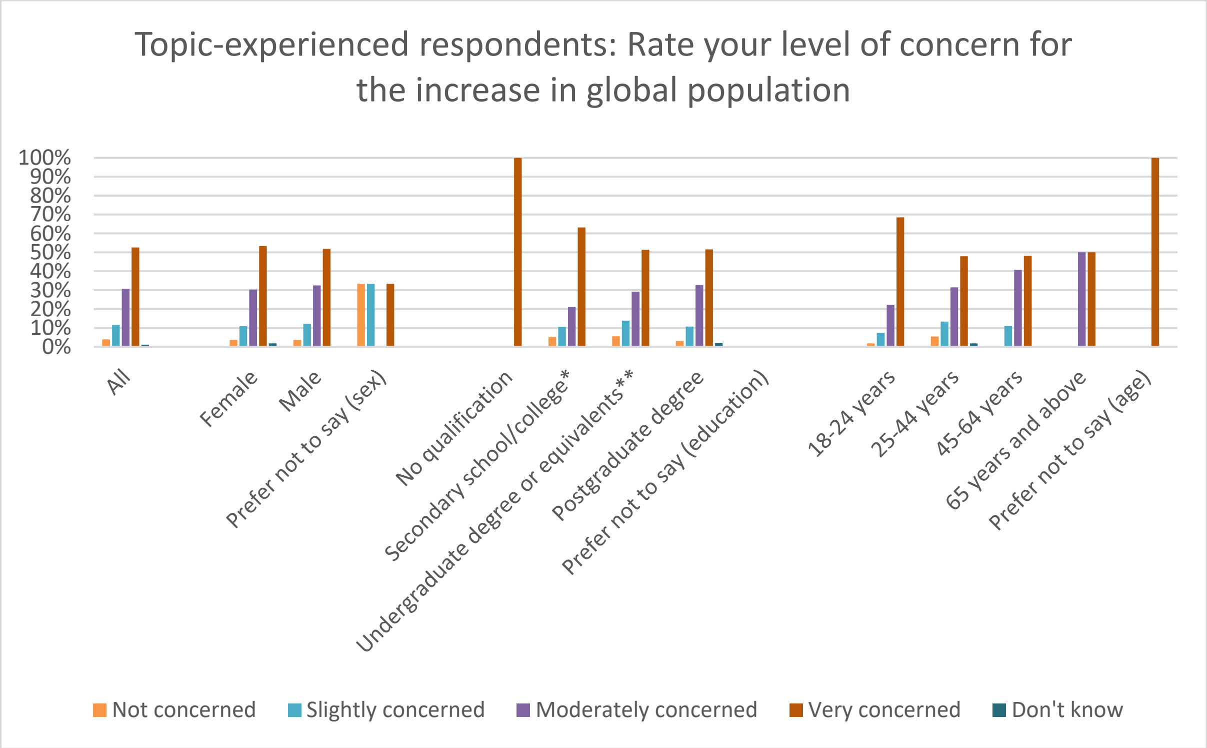

Figure S12 - Percentage distribution of responses of topic-experienced respondents by demographics (sex, education and age) for increase in global population,* (16-18 years) and equivalents, ** Including trade/technical/vocational training.

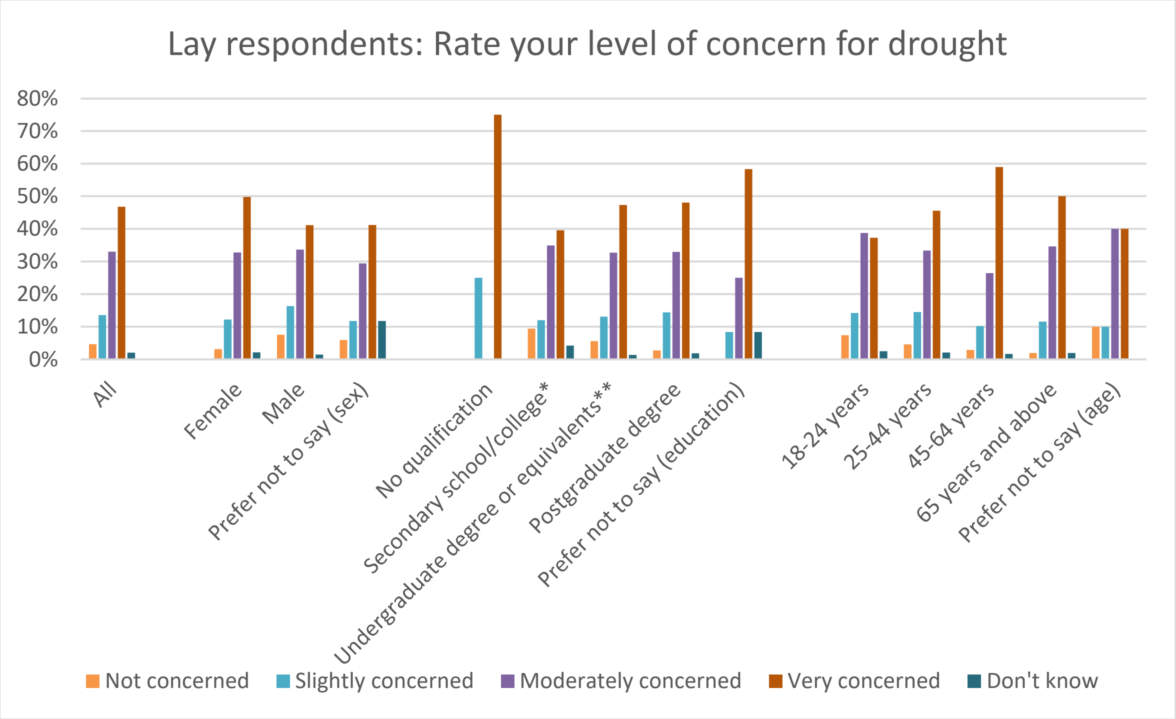

Figure S13 - Percentage distribution of responses of lay respondents by demographics (sex, education and age) for drought,* (16-18 years) and equivalents, ** Including trade/technical/vocational training.

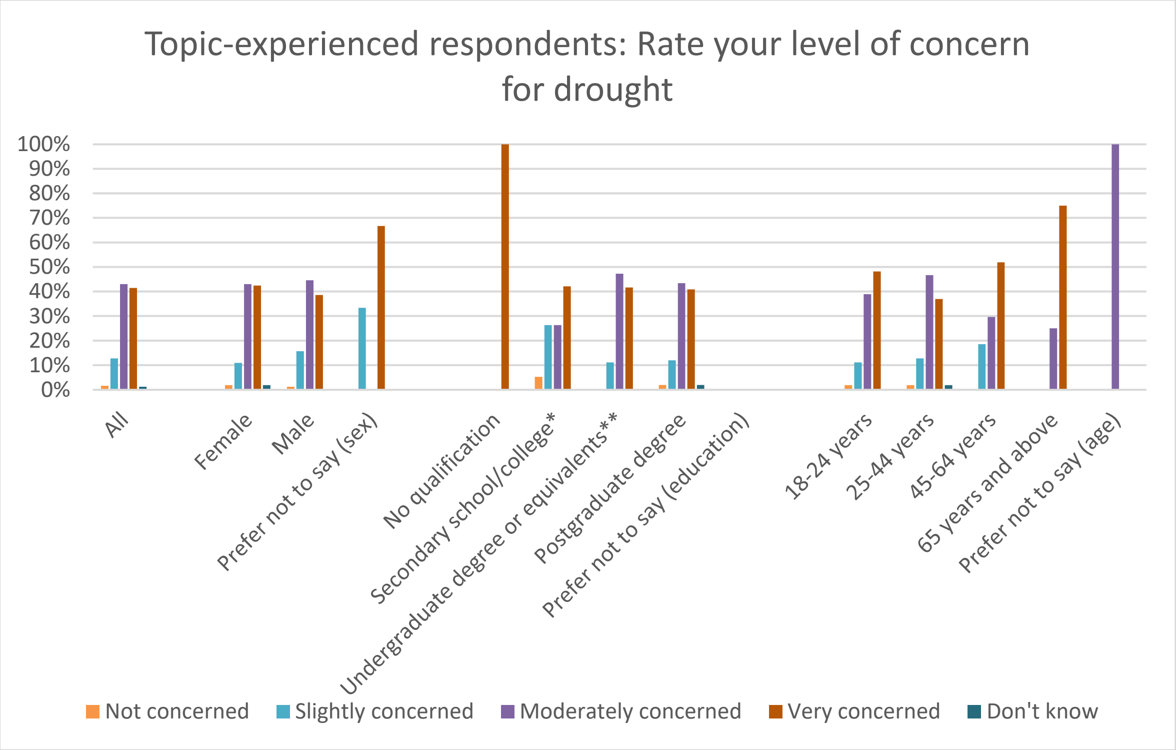

Figure S14 - Percentage distribution of responses of topic-experienced respondents by demographics (sex, education and age) for drought,* (16-18 years) and equivalents, ** Including trade/technical/vocational training.

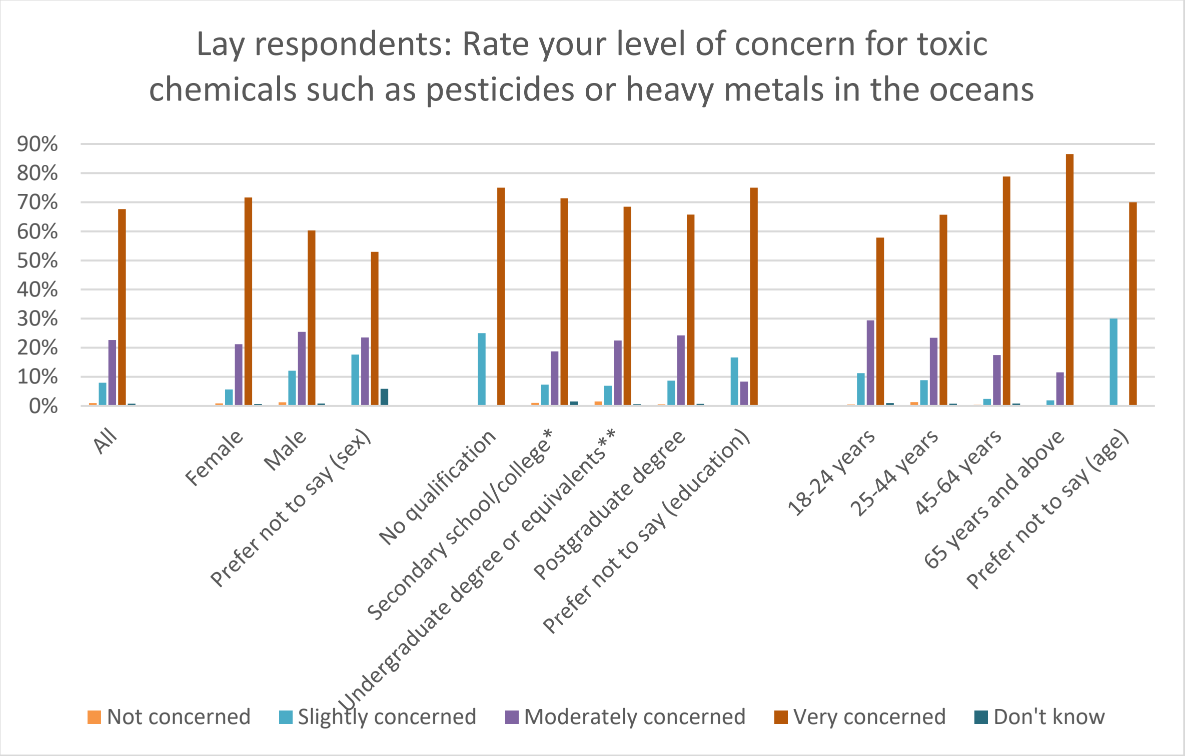

Figure S15 - Percentage distribution of responses of lay respondents by demographics (sex, education and age) for toxic chemicals,* (16-18 years) and equivalents, ** Including trade/technical/vocational training.

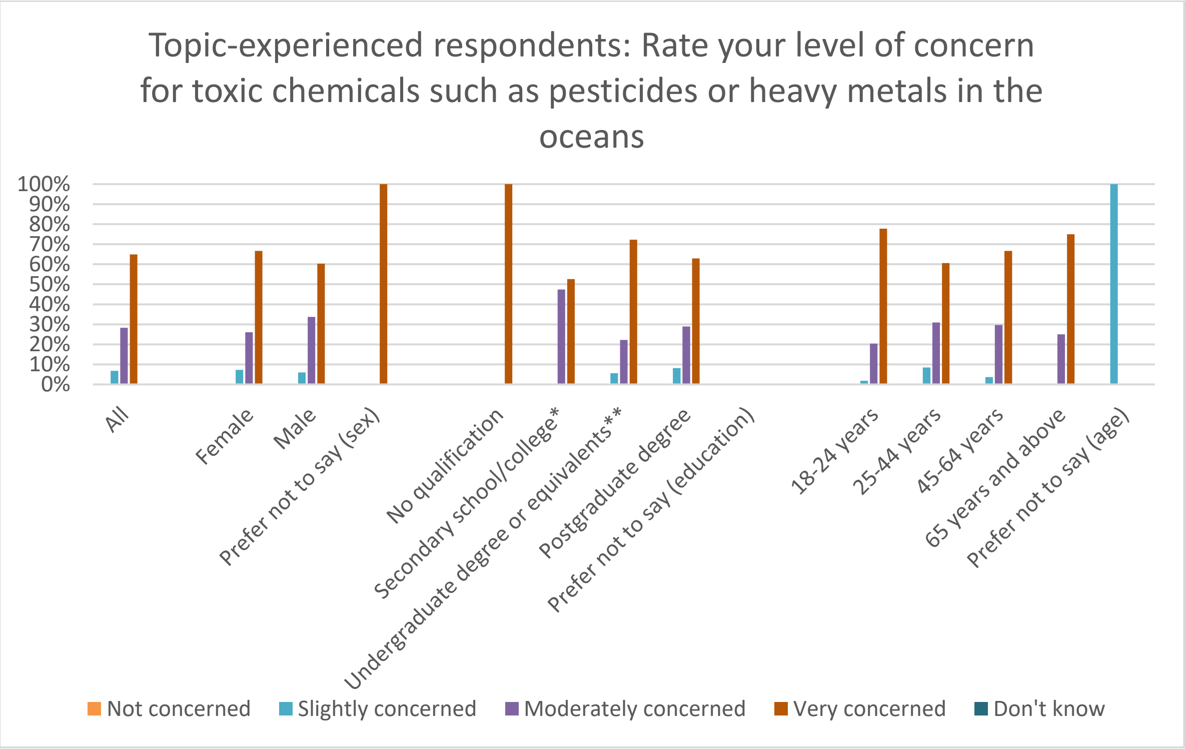

Figure S16 - Percentage distribution of responses of topic-experienced respondents by demographics (sex, education and age) for toxic chemicals,* (16-18 years) and equivalents, ** Including trade/technical/vocational training.

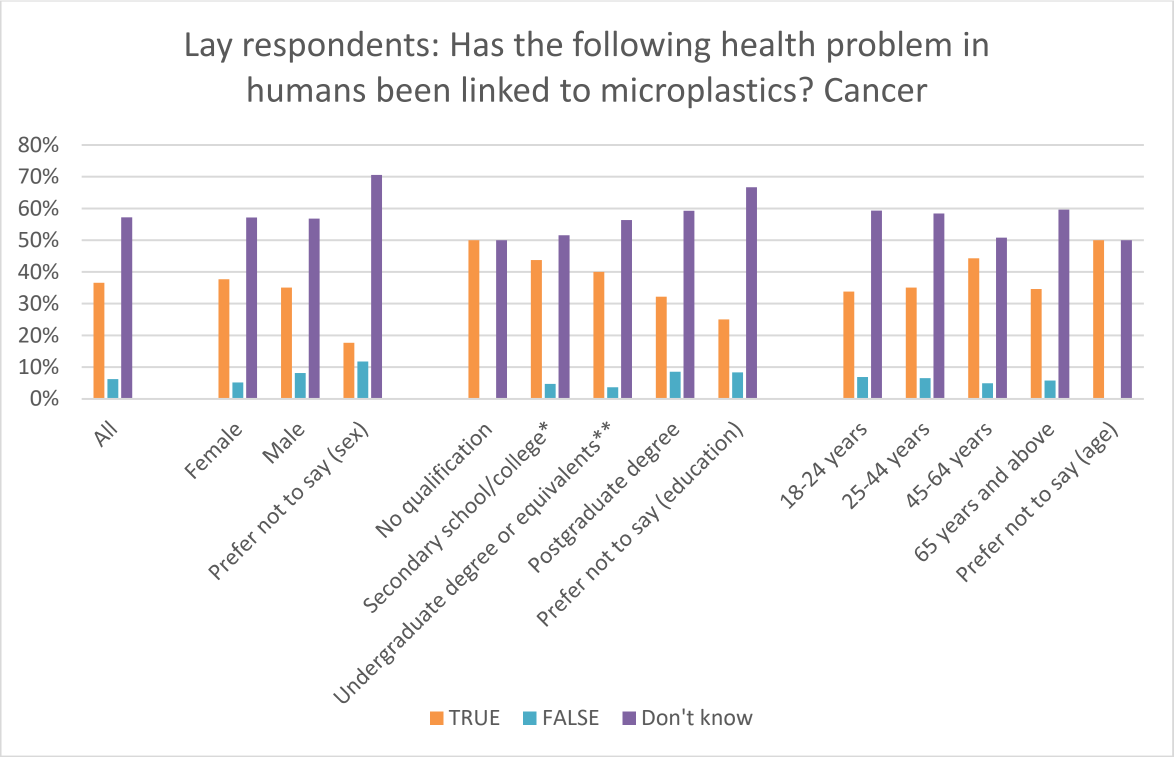

Figure S17 - Percentage distribution of responses of lay respondents by demographics (sex, education and age) for microplastics-cancer link,* (16-18 years) and equivalents, ** Including trade/technical/vocational training.

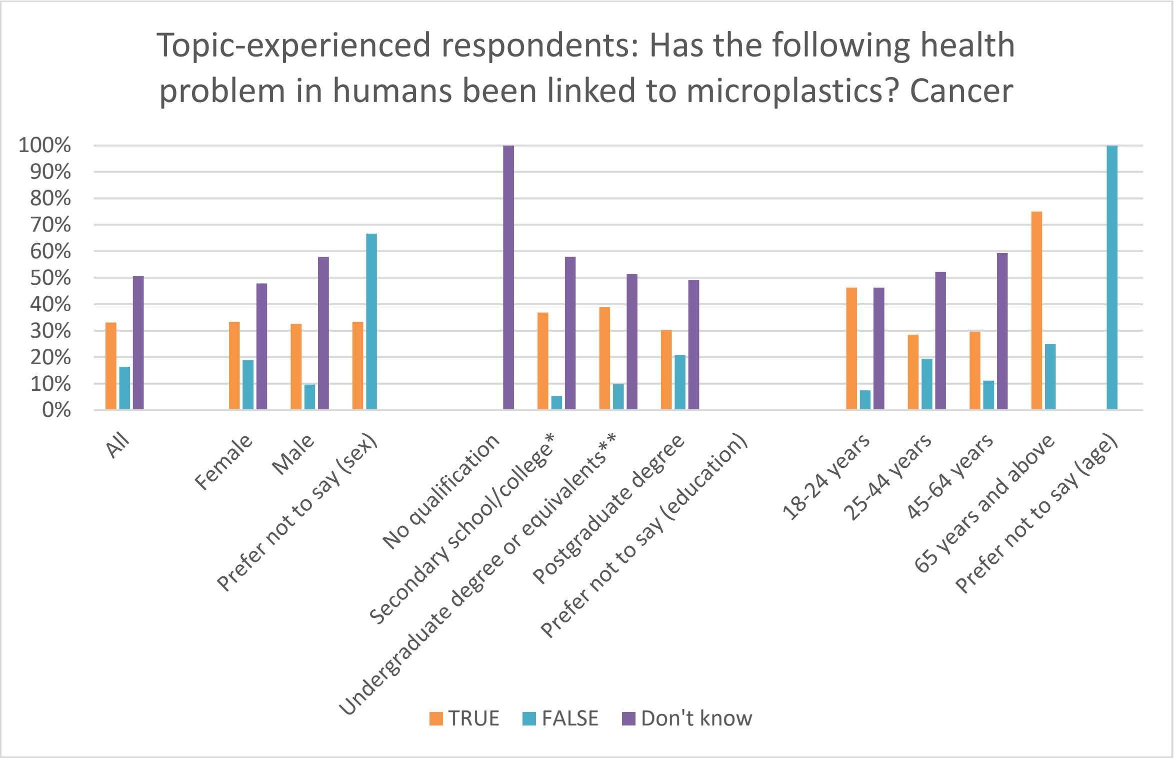

Figure S18 -- Percentage distribution of responses of topic-experienced respondents by demographics (sex, education and age) for microplastics-cancer link,* (16-18 years) and equivalents, ** Including trade/technical/vocational training.

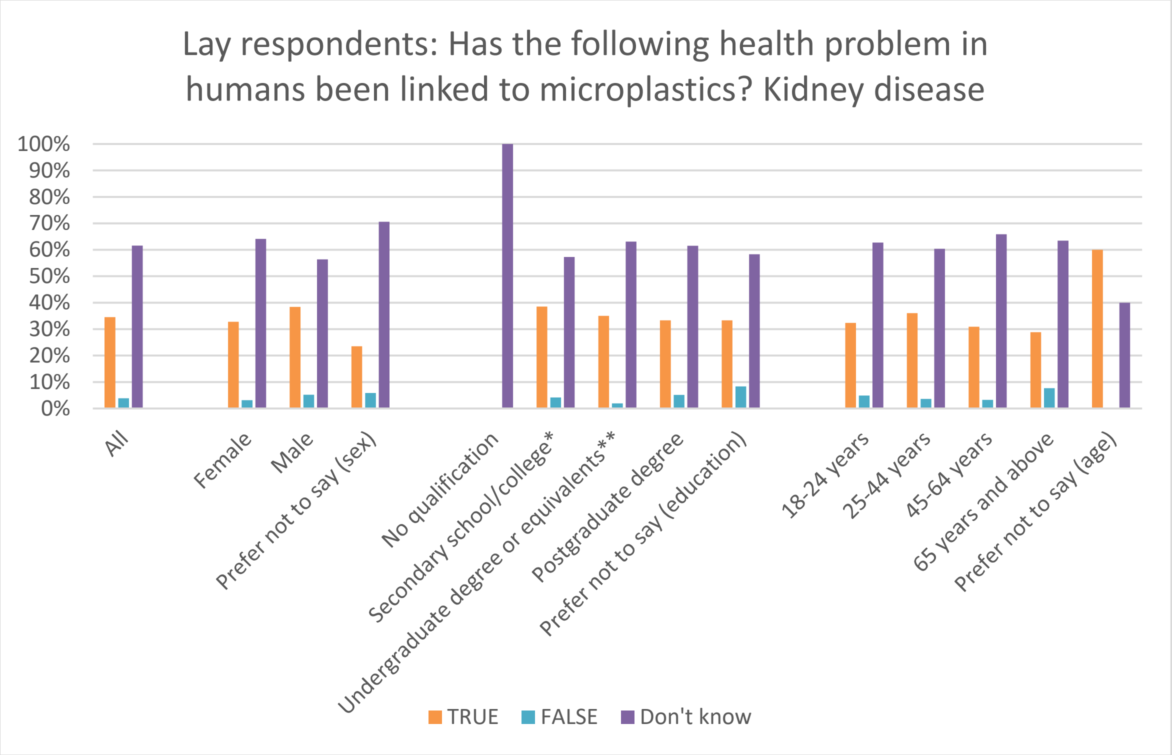

Figure S19 - Percentage distribution of responses of lay respondents by demographics (sex, education and age) for microplastics-kidney disease link,* (16-18 years) and equivalents, ** Including trade/technical/vocational training.

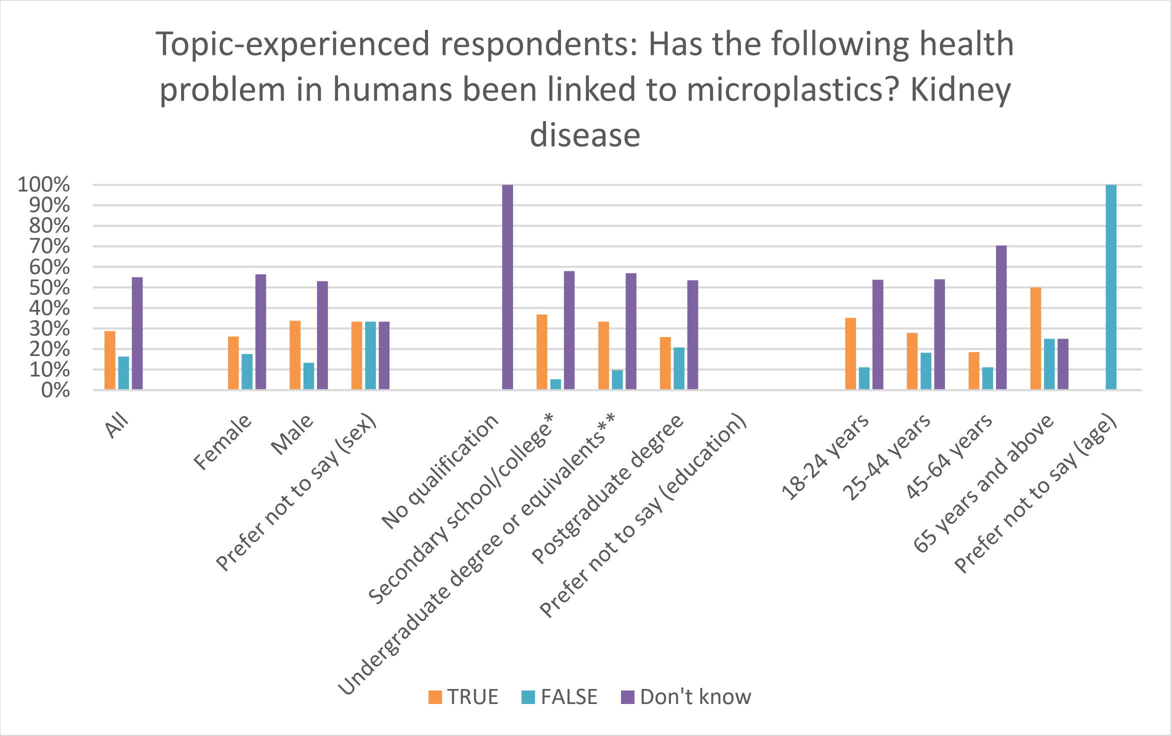

Figure S20 - - Percentage distribution of responses of topic-experienced respondents by demographics (sex, education and age) for microplastics-kidney disease link,* (16-18 years) and equivalents, ** Including trade/technical/vocational training.

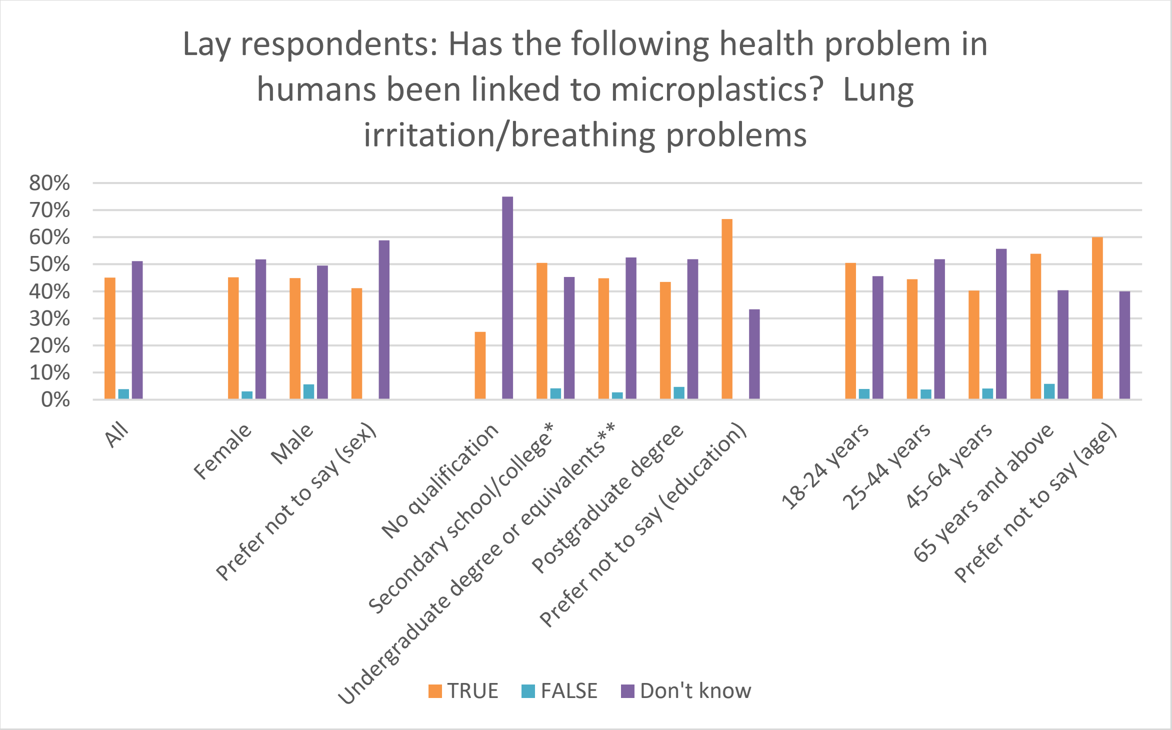

Figure S21 -- Percentage distribution of responses of lay respondents by demographics (sex, education and age) for microplastics-lung irritation/breathing problems link,* (16-18 years) and equivalents, ** Including trade/technical/vocational training.

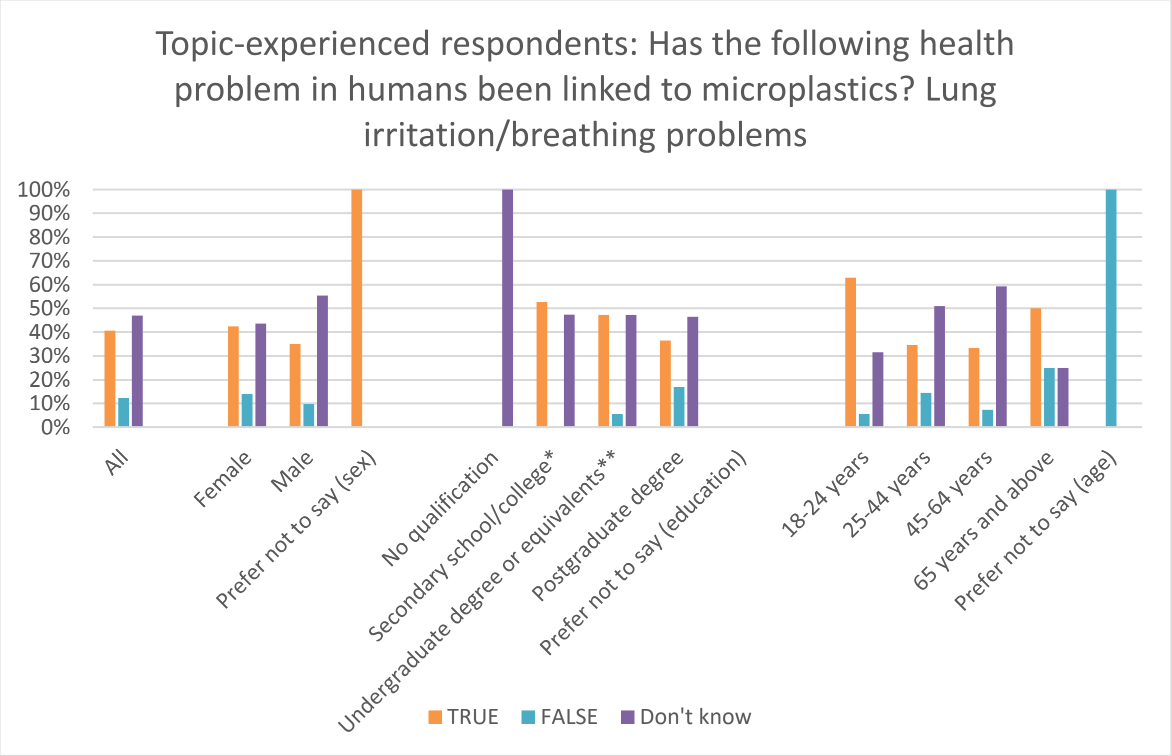

Figure S22 - Percentage distribution of responses of topic-experienced respondents by demographics (sex, education and age) for microplastics-lung irritation/breathing problems link,* (16-18 years) and equivalents, ** Including trade/technical/vocational training.

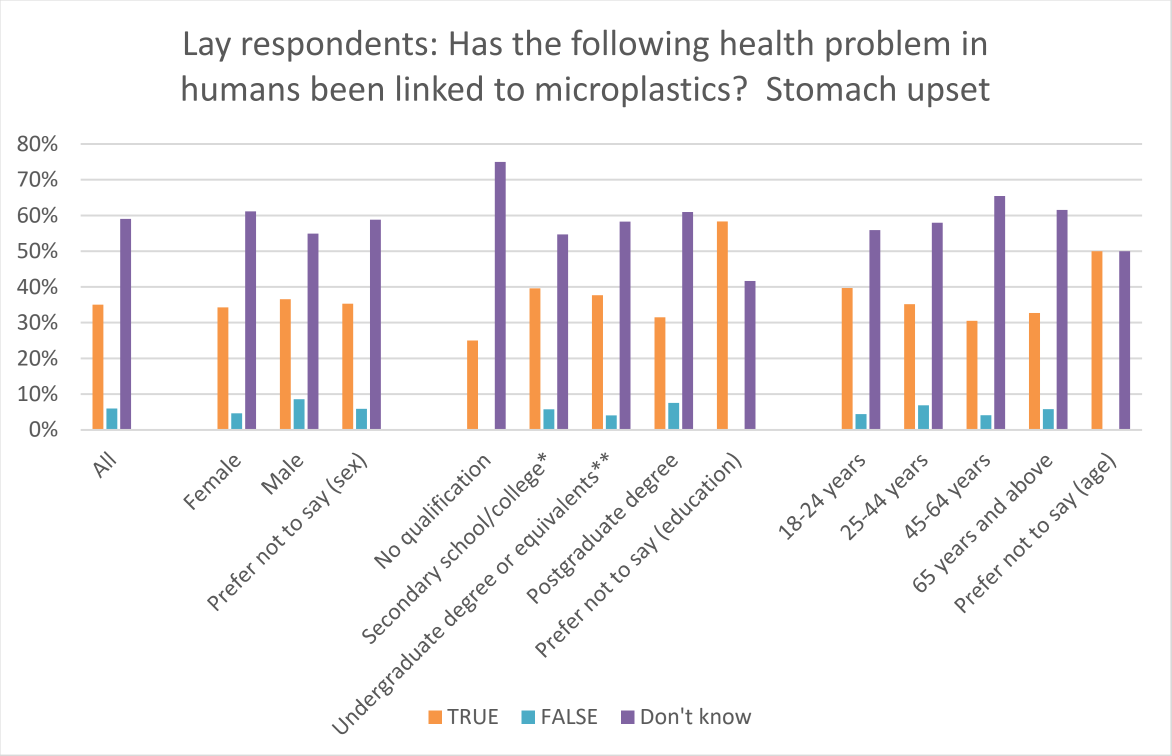

Figure S23 - Percentage distribution of responses of lay respondents by demographics (sex, education and age) for microplastics-stomach upset link,* (16-18 years) and equivalents, ** Including trade/technical/vocational training.

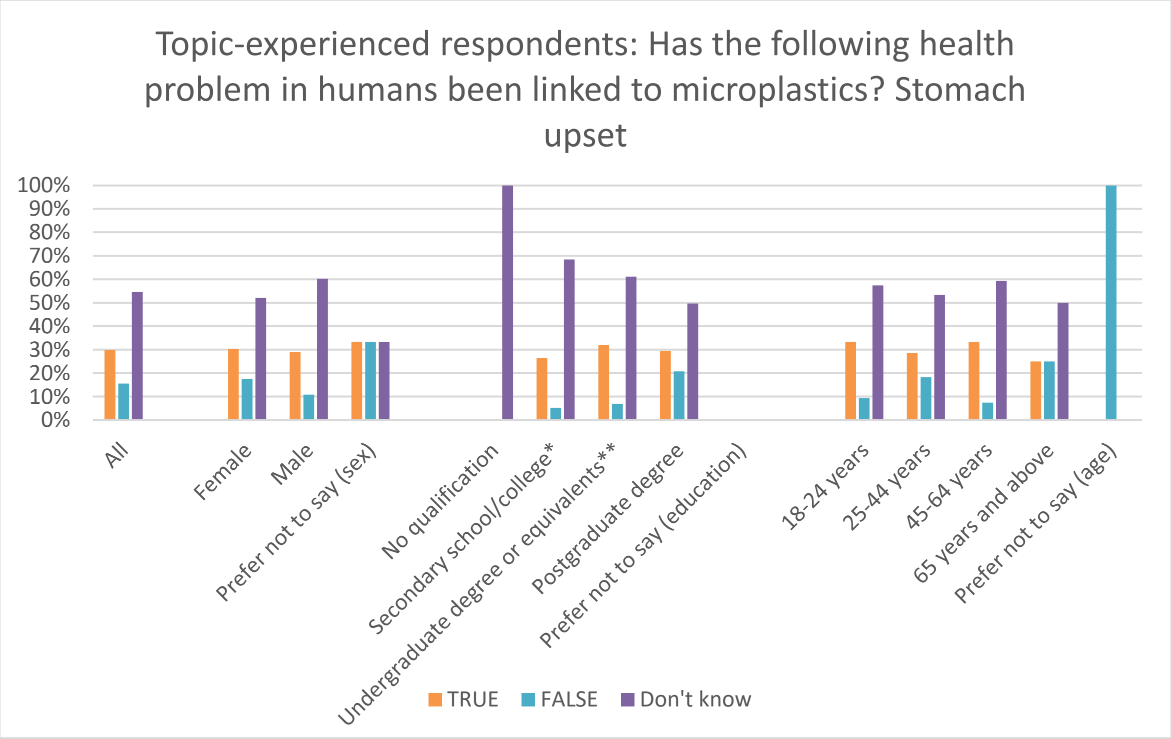

Figure S24 - Percentage distribution of responses of topic-experienced respondents by demographics (sex, education and age) for microplastics-stomach upset link,* (16-18 years) and equivalents, ** Including trade/technical/vocational training.

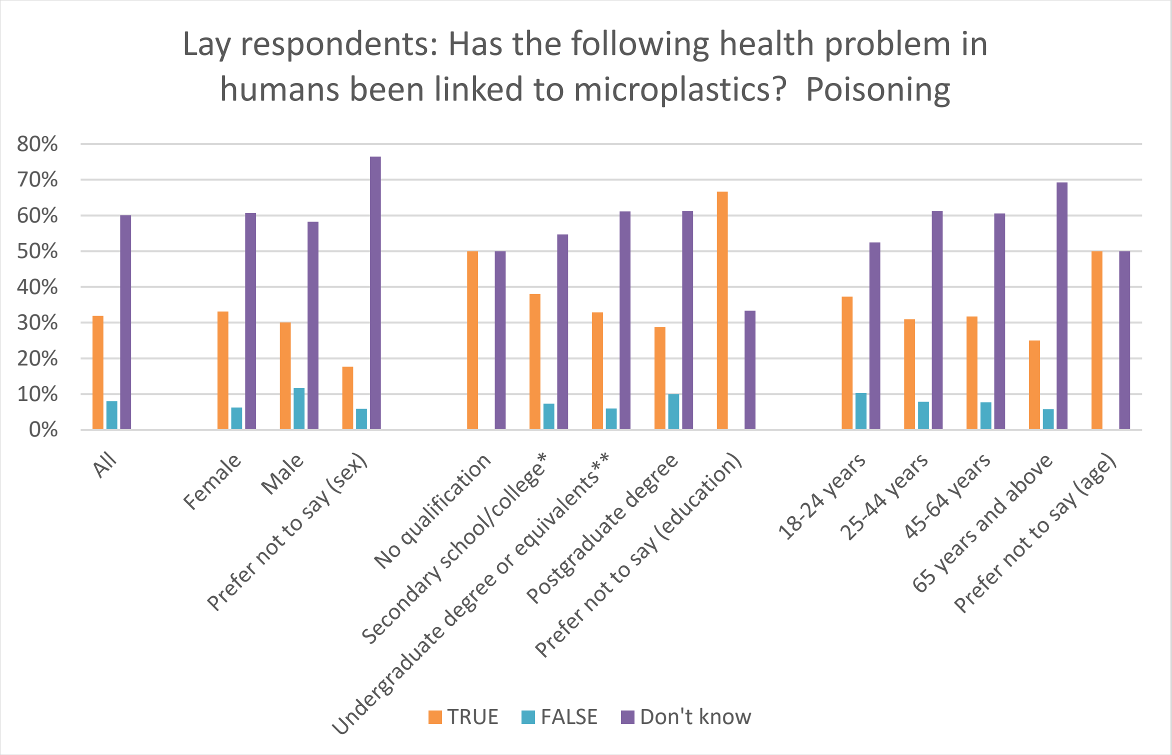

Figure S25 -- Percentage distribution of responses of lay respondents by demographics (sex, education and age) for microplastics-poisoning link,* (16-18 years) and equivalents, ** Including trade/technical/vocational training.

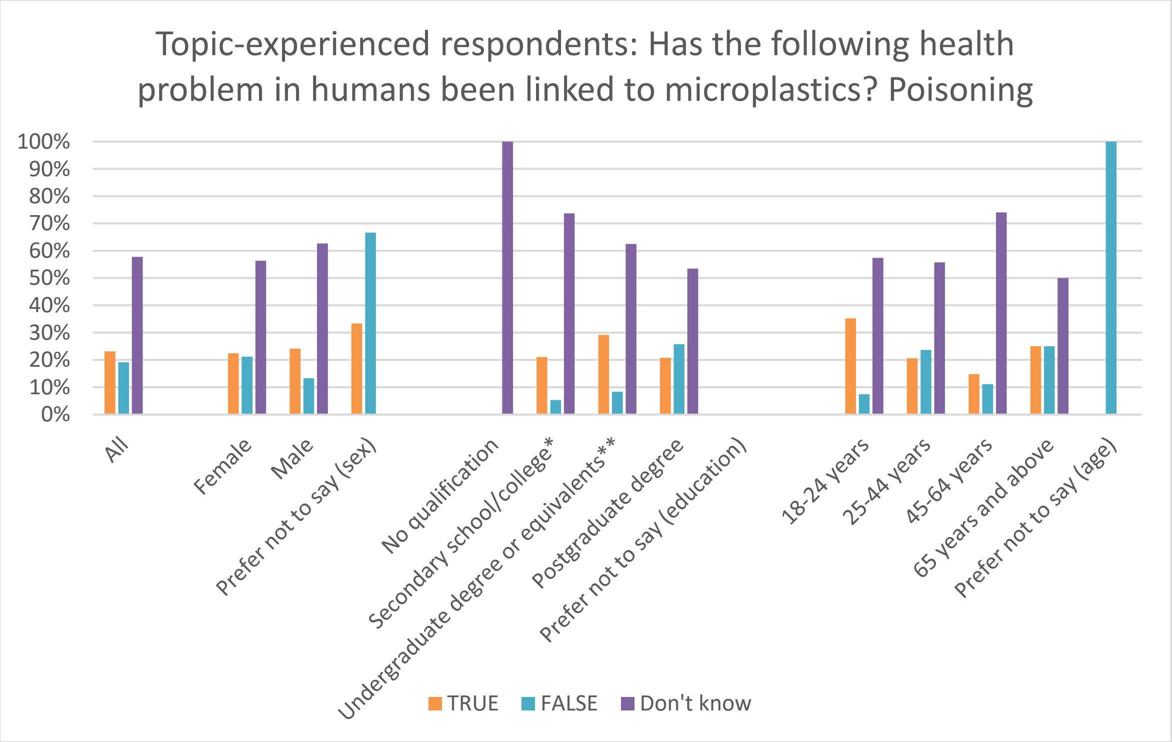

Figure S26 --- Percentage distribution of responses of topic-experienced respondents by demographics (sex, education and age) for microplastics-poisoning link,* (16-18 years) and equivalents, ** Including trade/technical/vocational training.

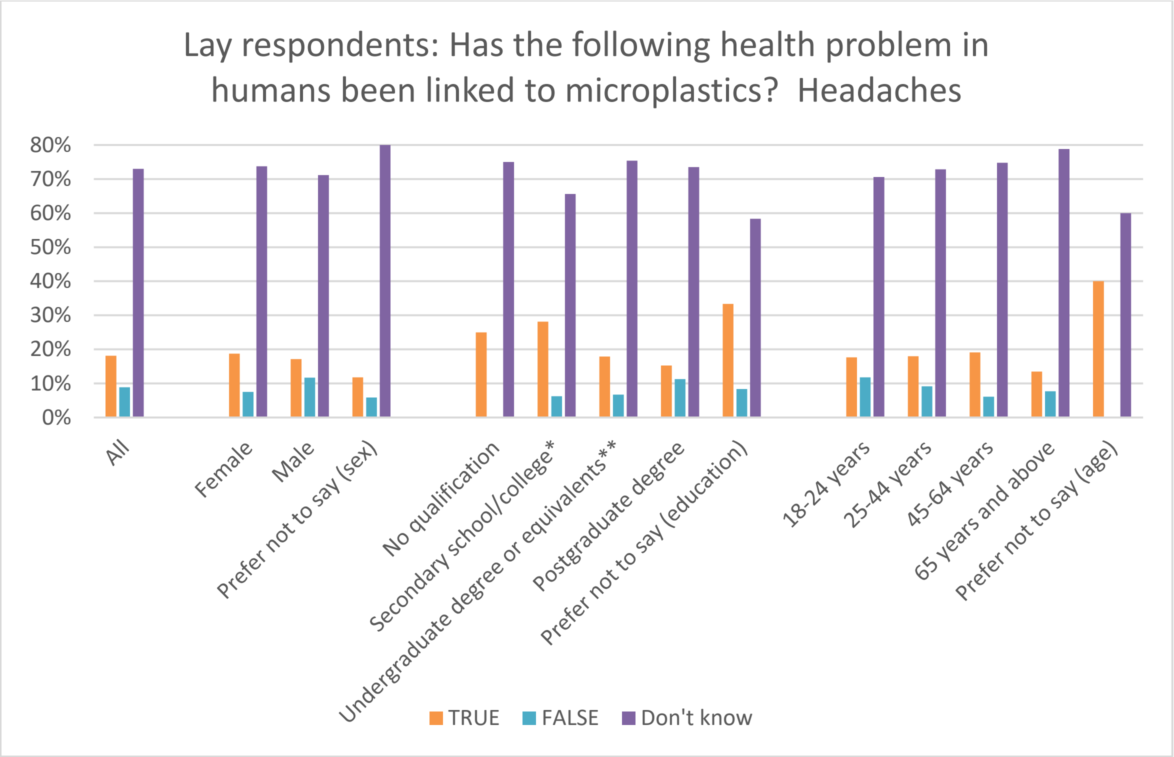

Figure S27 - Percentage distribution of responses of lay respondents by demographics (sex, education and age) for microplastics-headaches link,* (16-18 years) and equivalents, ** Including trade/technical/vocational training.

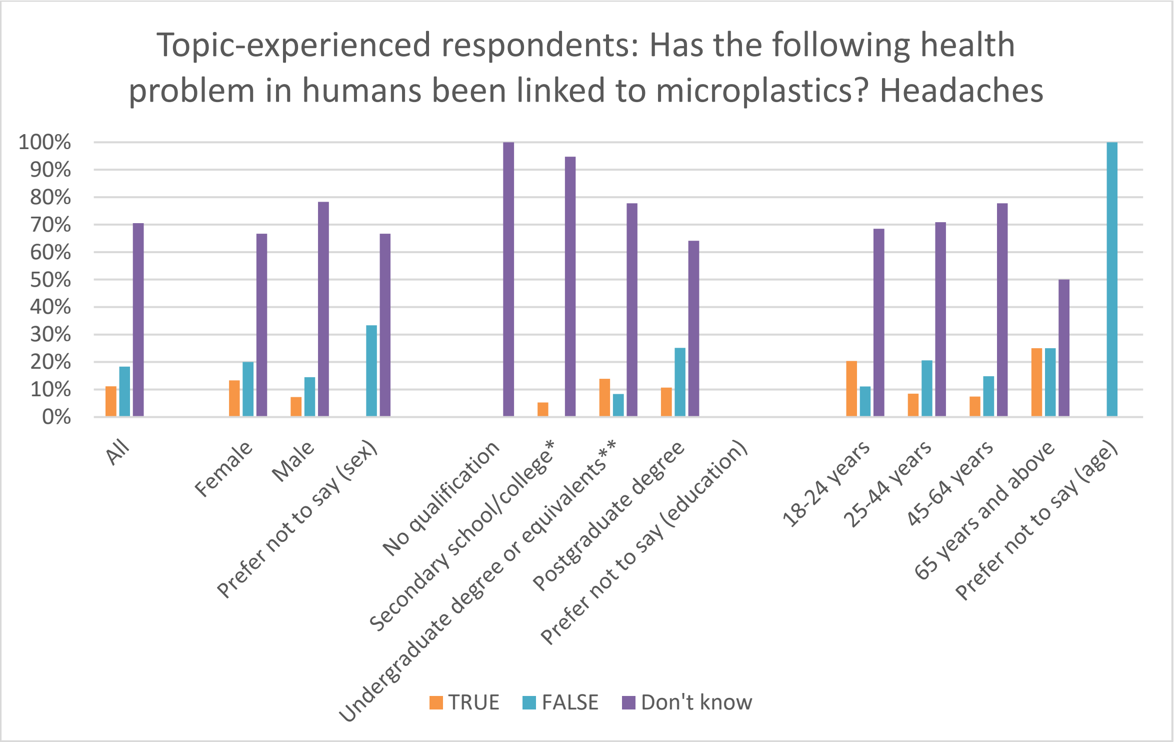

Figure S28 - Percentage distribution of responses of topic-experienced respondents by demographics (sex, education and age) for microplastics-headaches link,* (16-18 years) and equivalents, ** Including trade/technical/vocational training.

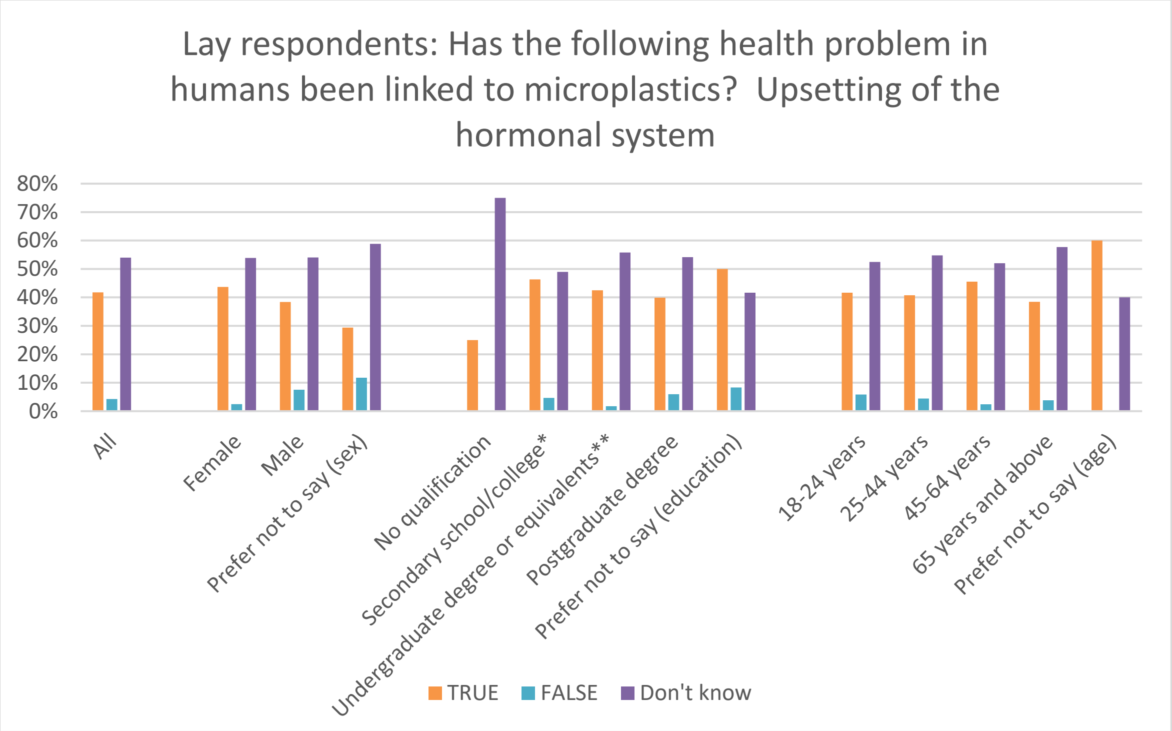

Figure S29 - Percentage distribution of responses of lay respondents by demographics (sex, education and age) for microplastics-upsetting of the hormonal system link,* (16-18 years) and equivalents, ** Including trade/technical/vocational training.

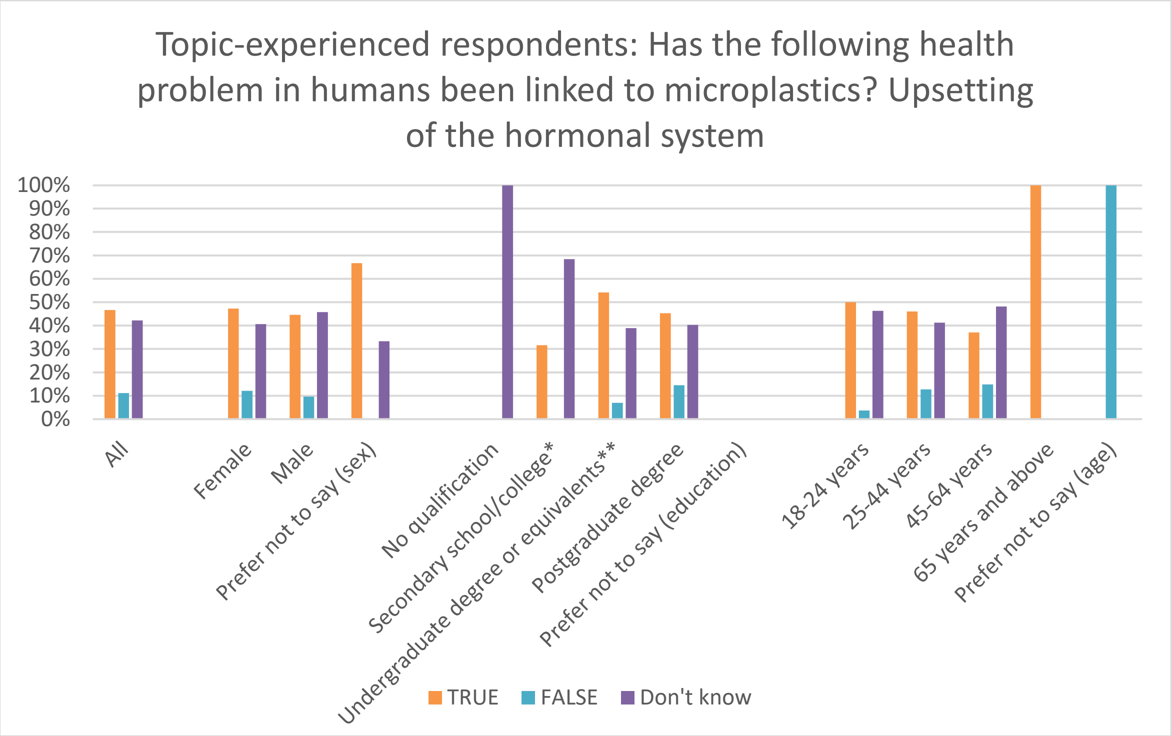

Figure S30 -- Percentage distribution of responses of topic-experienced respondents by demographics (sex, education and age) for microplastics-upsetting of the hormonal system link,* (16-18 years) and equivalents, ** Including trade/technical/vocational training.

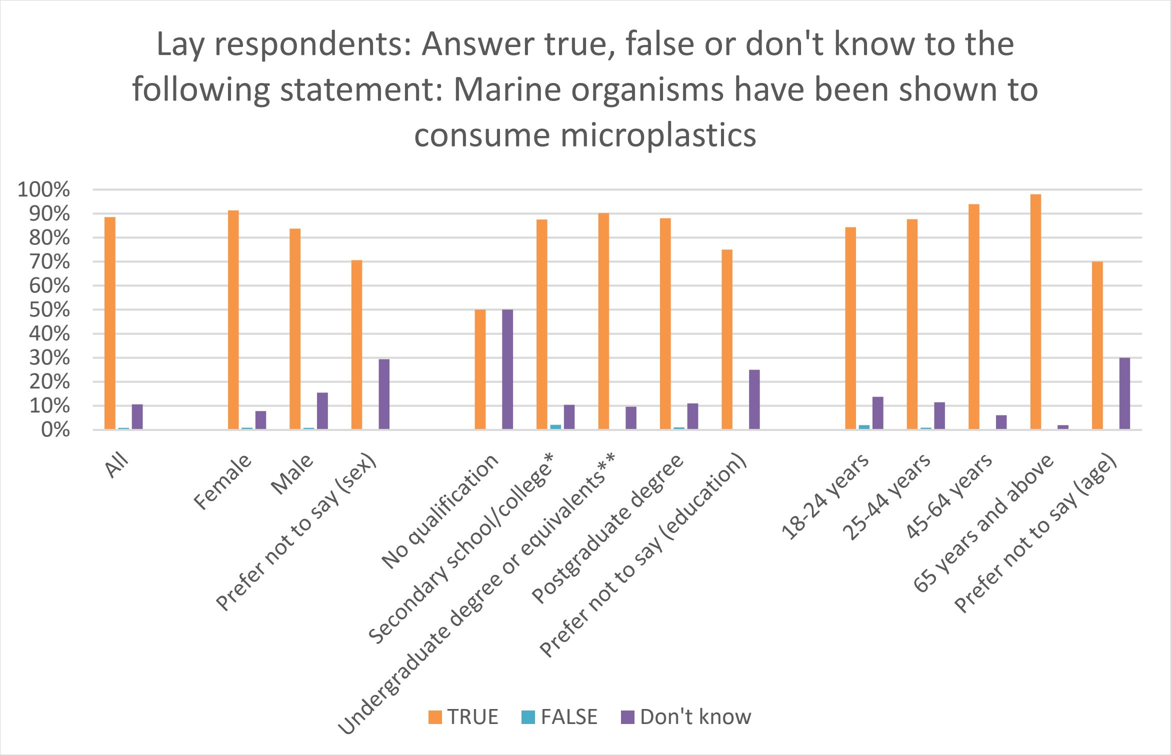

Figure S31 -- Percentage distribution of responses of lay respondents by demographics (sex, education and age) for 'marine organisms have been shown to consume microplastics',* (16-18 years) and equivalents, ** Including trade/technical/vocational training.

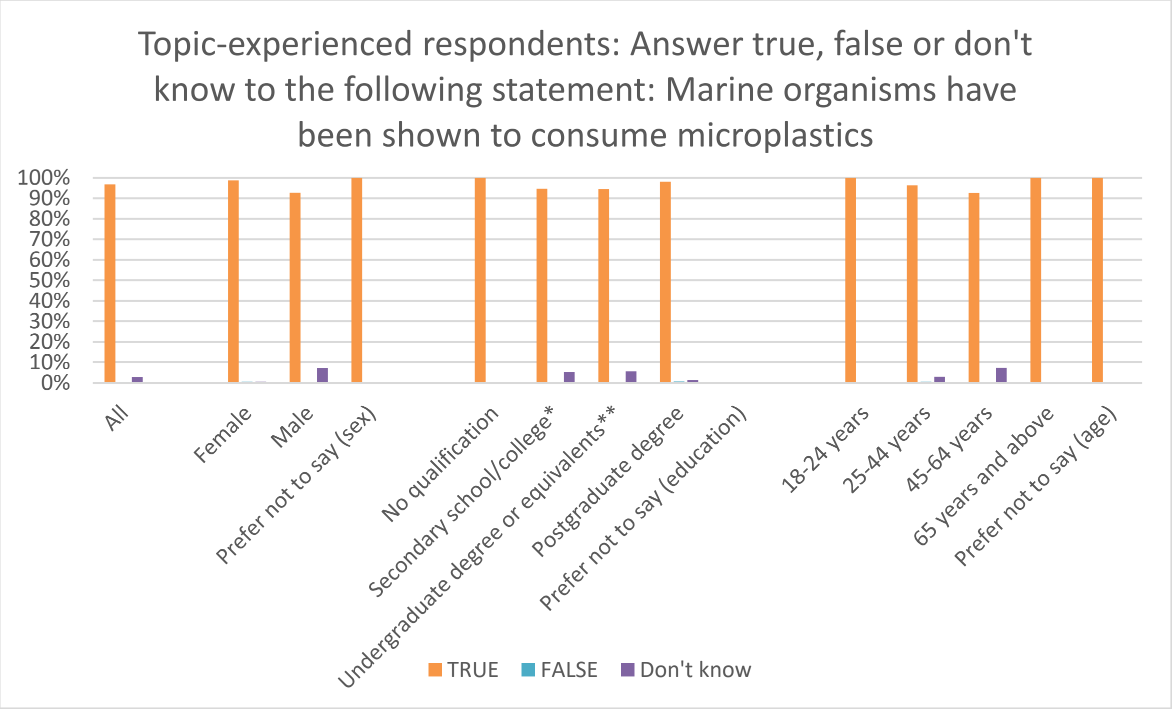

Figure S32 -- Percentage distribution of responses of topic-experienced respondents by demographics (sex, education and age) for 'marine organisms have been shown to consume microplastics',* (16-18 years) and equivalents, ** Including trade/technical/vocational training.

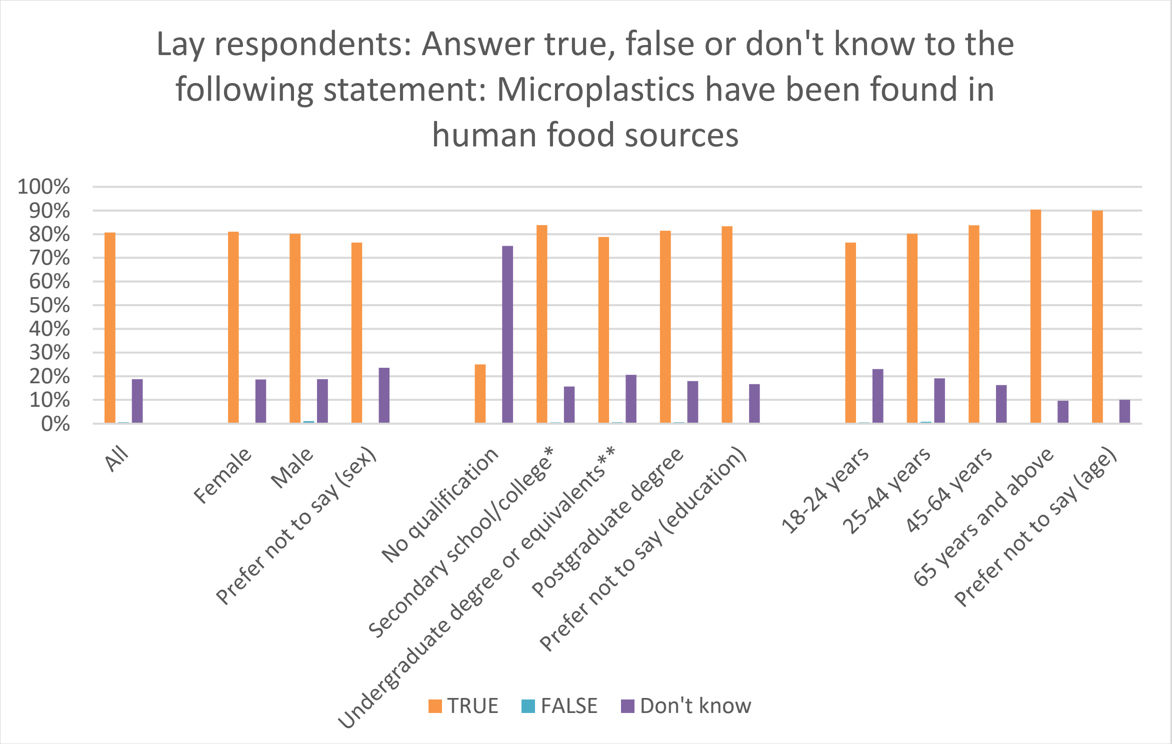

Figure S33 -- Percentage distribution of responses of lay respondents by demographics (sex, education and age) for 'microplastics have been found in human food sources',* (16-18 years) and equivalents, ** Including trade/technical/vocational training.

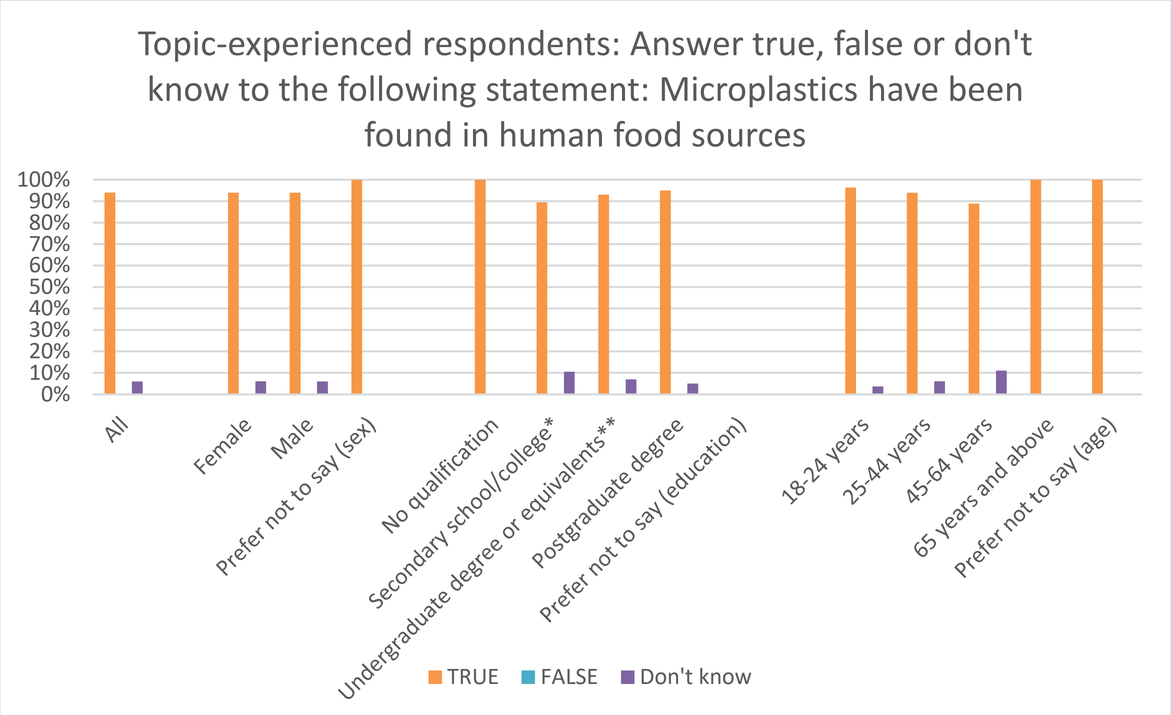

Figure S34 -- Percentage distribution of responses of topic-experienced respondents by demographics (sex, education and age) for 'microplastics have been found in human food sources',* (16-18 years) and equivalents, ** Including trade/technical/vocational training.

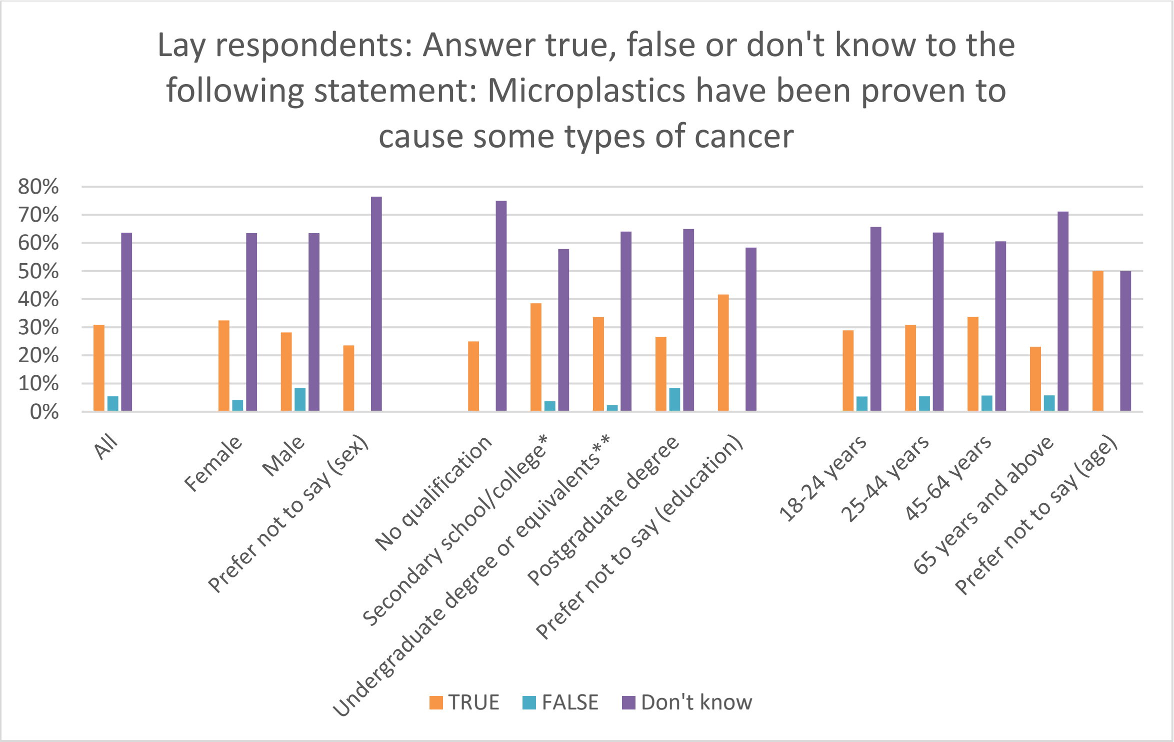

Figure S35 -- Percentage distribution of responses of lay respondents by demographics (sex, education and age) for ‘microplastics have been proven to cause some types of cancer’,* (16-18 years) and equivalents, ** Including trade/technical/vocational training.

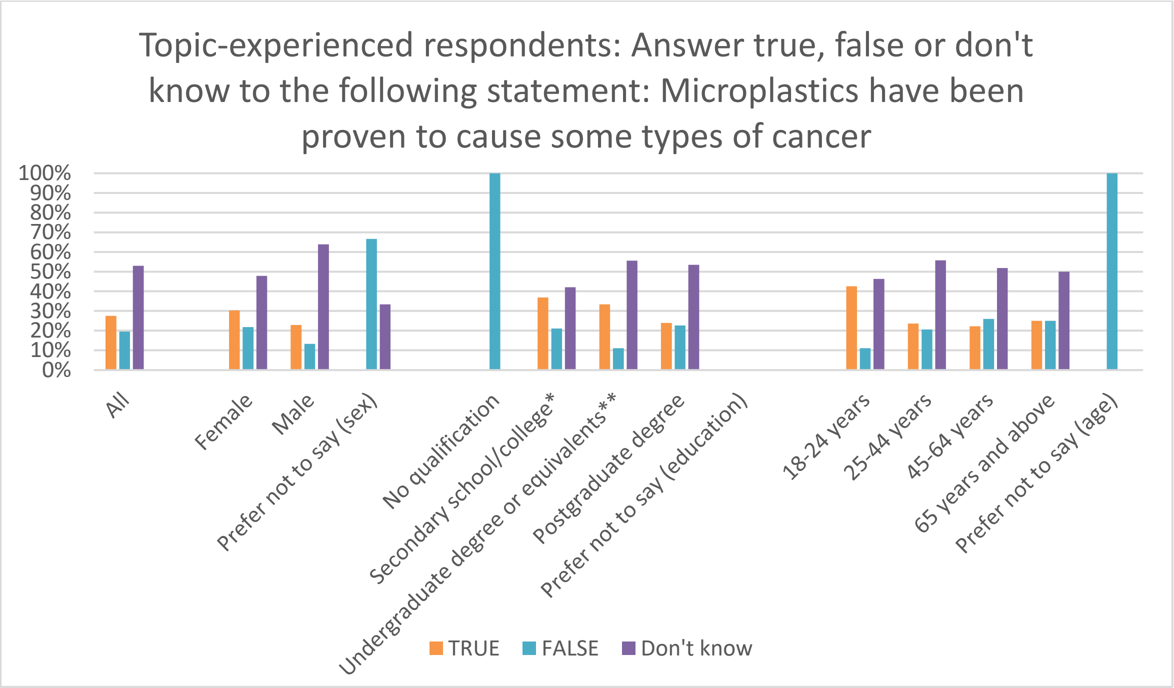

Figure S36 -- Percentage distribution of responses of topic-experienced respondents by demographics (sex, education and age) for ‘microplastics have been proven to cause some types of cancer’,* (16-18 years) and equivalents, ** Including trade/technical/vocational training.
